# Explaining disparities in robot applications among nations and regions: A cross-level lens of cultural tightness-looseness

**DOI:** 10.1371/journal.pone.0321173

**Published:** 2025-04-16

**Authors:** Jian Guan, Xiao-Ping Chen, Sitong Yu, Xin Qin

**Affiliations:** 1 Antai College of Economics and Management, Shanghai Jiao Tong University, Shanghai, China; 2 Michael G. Foster School of Business, University of Washington, Seattle, Washington, United States of America; 3 Nanyang Business School, Nanyang Technological University, Singapore; 4 Sun Yat-sen Business School, Sun Yat-sen University, Guangzhou, Guangdong, China; King Fahd University of Petroleum & Minerals, SAUDI ARABIA

## Abstract

In this study, we aim to explain the large disparities among countries and regions on industrial robot application in terms of robot density and robot growth. Based on the premise that people in all cultures have the same potential for innovation, we propose a cross-level lens of cultural tightness-looseness to predict that, in the nascent robotics industry where rules and regulations are underdeveloped (i.e., a loose domain), people in tight cultures (e.g., Singapore, Japan, China) are more likely to innovate than those in loose cultures (e.g., the UK, U.S., the Netherlands) because their creativity is permitted in this or a few other loose domains only. We test this theoretical lens using multi-source longitudinal archival data on robot applications. Several significant findings emerge. First, there is a significant positive relationship between cultural tightness and robot application across 32 countries and territories from 1993 to 2022 (Study 1). Second, such a positive relationship also appears across 50 states in the United States from 1998 to 2022 (Study 2a), and across 31 provinces in China from 2008 to 2022 (Study 2b). Finally, the interaction effect between country- and region-level cultural tightness on robot application is significant (Study 3). These findings provide strong empirical support for our cross-level lens of cultural tightness and shed light on how cultural tightness at different levels interacts to affect incremental innovation (i.e., robot application) in a loose domain (i.e., the robotics industry). Moreover, these findings suggest that loosening tight control on certain domains of a tight culture would remarkably boost creativity and incremental innovation in these domains.

## Introduction

Robots and many other types of artificial intelligence (AI) are revolutionizing the world in numerous areas, including politics (e.g., political audience diversity) [1], the economy (e.g., labor markets) [2], society (e.g., religion) [3], and technology (e.g., medicine) [4]. In 2022, a record 3.9 million industrial robots were operating across the globe (International Federation of Robotics, henceforth IFR) [5]. In Asian countries such as Singapore, Japan, and China, social robots have also been used in settings like hospitals, hotels, restaurants, homes, and even churches and temples [6–8]. Meanwhile, robot application shows large disparities across countries [7,9,10]. A recent report by IFR indicates that since 2017, China has experienced the fastest growth in industrial robot application in the world, with a staggering 300% increase in robot density per capita, reaching 392 units per 10,000 employees. In contrast, robot density in the United States (U.S.) and the United Kingdom has only seen modest growth, increasing by less than 43% and 16%, with 285 and 98 units per 10,000 employees, respectively [11].

The surprising trend of disparity in robot application between the above-mentioned countries and regions presents an important phenomenon that begs theoretical and empirical explanations. Viewing robot application as an incremental rather than radical innovation, it is imperative to ask: why does incremental innovation seem to be more prevalent in China than in the U.S.? An extensive literature review reveals a myriad of complex factors that may answer this question [7]. However, we submit that a particularly promising perspective is a cultural lens, specifically, the theoretical lens of cultural tightness-looseness. *Cultural tightness-looseness* refers to the strength of social norms and tolerance of deviance from norms in a culture [12–14]; a tight (vs. loose) culture has stronger social norms and stricter sanctions for deviant behaviors. Accumulated research has demonstrated the potential influence of cultural tightness-looseness on creativity and innovation, with the general consensus that individuals from tight cultures have narrower behavioral options, higher concerns about being sanctioned for deviance, and are more resistant to change, and thus they are less likely to engage in creative and innovative activities than do those in loose cultures [12,15].

These findings imply that people in tight cultures tend to be more conservative and less willing to adopt newly developed technologies such as industrial or service robots, as doing so inherently entails change and risk [16]. However, since Singapore, the Republic of Korea, Japan, and China are tight cultures, whereas the United States, Italy, Spain, and the United Kingdom are loose cultures according to several large sample studies [12,17], the previously mentioned robot application data [18] seem to contradict what would have been predicted by the theory of cultural tightness-looseness.

To add to that, a similar pattern of results – i.e., culture tightness was not negatively but positively related to incremental innovation, as represented by patent applications – was obtained by Chua et al. [19], who conducted their study within China. Specifically, they found that the tight provinces/cities (e.g., Beijing, Shanghai, Guangdong) showed more incremental innovation than those less tight provinces/cities (e.g., Chongqing, Guizhou, Hunan), even after controlling for a bunch of potential influencing factors such as GDP per capita, education spending per GDP, and research and development (R&D) spending per GDP. To justify that these findings were in alignment with the theory of tightness, the authors explained that incremental innovation actually reflected normative rather than innovative behavior, because the Chinese government’s push for innovation facilitated a ‘norm of innovation’ in the tight provinces/cities.

**Fig 1:**
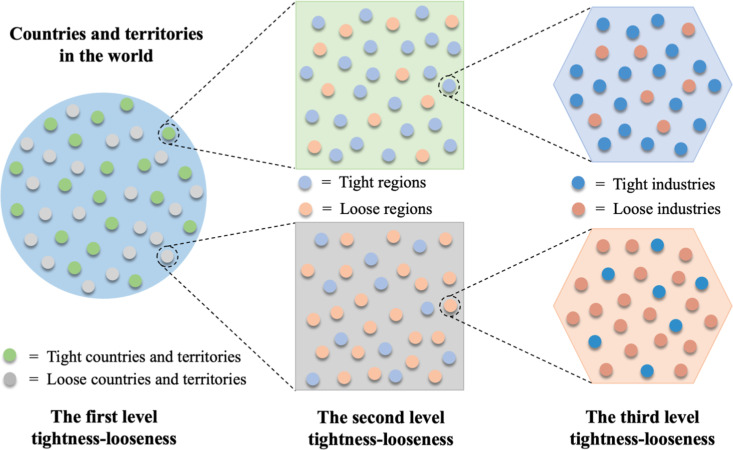
A cross-level lens of cultural tightness-looseness. There are tight countries and territories (green spots; e.g., China) and loose countries and territories (gray spots; e.g., the U.S.) in the world (blue earth). A tight country or territory has stronger social norms and stricter sanctions for deviant behavior, as reflected by the prevalence of tight regions (light blue spots), while there are also some loose regions (light orange spots). The third level is the industry level, which includes tight industries (dark blue spots; e.g., financial industry) and loose industries (dark orange spots; e.g., robotic industry).

Although this justification is somewhat reasonable, it is rather stretching. In this paper, we offer a more logical theoretical account by looking beyond one level of culture tightness to explain the seemingly contradictory phenomenon of industrial robot application. This theoretical account is called a cross-level lens of cultural tightness, which posits that multi-level tightness should be considered simultaneously to predict creative or innovative behaviors in a specific domain. In particular, we articulate that country-level tightness is determined by the tightness of many spheres (e.g., states/provinces) within that country, which in turn is determined by the tightness of various domains (e.g., industry) within the state/province. Fig 1 illustrates a three-level relationship between the country- and region-level tightness-looseness and the third industry-level tightness-looseness.

We further borrow Meyer et al.’s [20] definition of situational strength to conceptualize the second- or third-level tightness, referring to it as domain tightness -- the extent to which there exist implicit or explicit rules and norms regarding the desirability of potential behaviors in a specific domain [21]. It can be seen in Fig 1 that within a tight country, although many states/provinces have explicit or implicit rules and structures to restrict behaviors, there exist loose states/provinces that have few rules that allow flexibility and freedom. For example, within China, some provinces are looser than others [19], and some industries (e.g., movie and social media) have stricter regulations than other industries (e.g., e-commerce or new energy). Likewise, in loose cultures where few restrictions are applied in many domains, there exist domains that have clear rules and severe punishment for rule-breaking behaviors. For instance, in the U.S. where the culture is generally loose, inner states are tighter than coastal states [13], and manufacturing companies have much tighter human resource policies than high-tech companies [20,22].

It should be noted that the third level is nested within the second level, which is nested within the first level, and that the levels can be extended to a larger number, depending on the analytical needs. In this paper, we set the first level at the country level, the second at the region/state/province level, and the third at the industry level [21]. The cross-level lens of tightness-looseness can be used to predict the effect of the first level (i.e., country) or second level (i.e., state/province) tightness on incremental innovation (i.e., robot applications) at the third level (i.e., the robotics industry); it can also be applied to predict the interactive effects of the first level and second level tightness on robot application at the third level.

Our research makes several contributions to understanding when and where human innovative behaviors flourish from a cultural perspective.

1. It extends the theory of cultural tightness by proposing a cross-level lens. The existing theory of cultural tightness takes a uni-level approach [13], leaving contradictory findings difficult to explain. We go beyond the first level (i.e., country) to include the second and third-level domain tightness to capture the cross-level interaction effect, offering novel theoretical insights into how cultural tightness functions at multiple levels to influence innovative behaviors.
2. Our findings challenge the basic assumption that cultural tightness is related to restricted behaviors and limited activities [23]. Instead, we suggest that the relationship between cultural tightness and innovation is not always negative [12,13,19]; it is contingent on domain tightness. In loose domains such as the robotics industry, people in tight cultures are in fact more actively engaged in innovative activities, resulting in higher levels of robot application, both across and within countries.
3. Our findings contribute to innovation research by providing valuable insights into how cultural tightness may ‘facilitate’ domain innovation. We suggest that domain looseness is directly related to people’s engagement in innovative activities (i.e., robot application), the looser a domain is, the higher the level of innovative activity, while higher-level cultural tightness could amplify this effect.

## Theoretical grounding and hypothesis development

### The robotics industry: A loose domain in an economy

Compared to traditional industries like medical devices [24] that have strict rules and regulations on multiple levels of practice, the robotics industry seems to enjoy more freedom at this stage of progress due to its rapid development, manifested in areas such as robot-related production, installation [11], and investment [25] activities. For example, during the past decade, the operational stocks of industrial robots have surged to 3.903 million units in 2022, representing a 2349.78% increase from 2012, with the top 10 nations sharing over 85% of all robots [18]. The amount of global corporate investment in AI also reached 189.59 billion U.S. dollars in 2022, 13 times that of 2013 [25]. In other words, the robotics industry in many countries is still nascent, emerging, and in flux.

Because of its rapid development, the rules and regulations in the robotics industry are underdeveloped and evolving. Global regulation of robots and AI is still in fragmented early stages [25,26], lagging behind the development of technology itself. An extensive review shows that 88% of the 84 documents providing ethical principles or guidelines [27] for AI were released only after 2016 [28]. For example, the United Nations Educational Scientific and Cultural Organization (UNESCO) produced the first global standard on AI ethics, the *Recommendation on the Ethics of Artificial Intelligence*, in November 2021 [29], and the first comprehensive law on AI proposed by a major regulator, *the Artificial Intelligence Act*, was announced by European Commission (EU) only recently in June 2023 [30]. Research in AI global governance is also rather scant, primarily focusing on the development of AI ethics principles and reviewing existing initiatives [27,31].

Although governments have started to incorporate robots and AI in national strategies and legislative records, a consensus on robots and AI regulations remains elusive [32]. There are increasing calls for reassessing the effectiveness of current governance approaches and exploring innovative ones that are specifically tailored to address the unique uncertainties and complexities associated with robots and AI [25,30]. Overall, there is still ample room for the development of, and research into, robot- and AI-relevant regulatory laws, regulations, and bills.

As such, we construe the robotics industry as a loose industry in any country or economy. However, due to the differences in country-level cultural tightness, we suggest that the meaning of looseness may be more pronounced in a tight versus loose country/region. That is, in a typically tight country where the majority of industries are bound by stringent rules and regulations, a loose industry stands out as it presents many more opportunities for innovative activities compared to its counterpart in a typically loose country, where the majority of industries have less strict rules and regulations. In other words, as most industries in a tight economy do not allow much innovation, the robotics industry is likely to witness a surge of creativity instead.

### Cultural tightness and robot application

Industrial robot application is viewed as incremental rather than radical innovation because it does not represent groundbreaking inventions from 0 to 1 [33]; instead, it typically involves the use of advanced technologies (e.g., AI, sensors, novel materials, and automation) to address real-world challenges at the forefront of Industry 4.0 and smart factory implementation [11,34]. Robot application requires reimagining and optimizing workflows, experimentation, customization, adaptation, and integration, all of which are key components of incremental innovation [35]. More importantly, a recent study mapped and analyzed the global innovative landscape of robots and AI, and revealed a tremendous increase in patenting activities related to robots and AI since 2013, with a significant boom in 2015–2016 [36]. These findings suggest that robot application is closely intertwined with innovation.

Although previous studies have documented that innovation is more pervasive in loose than in tight cultures [13,15,37], as mentioned earlier, Chua et al. [19] found that incremental innovation, such as patent application, was more profound in tight than in loose cities/provinces in China, which was the opposite to previous findings. One of the main reasons, we reckon, is that previous research overlooked the domainial strength in which their prediction resided. For example, if the focused domain is a tight one in a tight culture (such as the movie industry in China), then innovation will be much weaker in a tight culture compared to a loose culture where the movie industry is NOT a tight one. While some scholars have begun to pay attention to domain tightness within a culture [21], there lacks clear delineation regarding the relationship between cultural tightness at the domain level and creativity manifestation. For instance, Yong et al. [21] conducted a three-level hierarchical meta-analytic regression analysis and found that within tight cultures, the relationship between each component of creativity (i.e., domain-relevant skills, creativity-relevant skills, and task motivation) and creativity was stronger compared to that within loose cultures. Chua et al. [38] conceptualized cultural tightness as consisting of formal versus informal domains, and explored how the strength of norms in formal and informal domains influenced employee behaviors. Farmer et al. [39] made a similar attempt to distinguish between different domains of creativity – idea generation versus idea realization, and suggested that in the idea realization domain, people in tight cultures perform better than those in loose cultures.

These studies suggest that attention to domain tightness is a useful approach to explaining the link between cultural tightness and innovative activities in a specific domain. We thus propose a cross-level lens of cultural tightness to predict the pattern of disparity in robot application between nations and regions [21]. Based on this cross-level theoretical lens, a critical reason for the increasing robot application in tighter nations and provinces is that robot application represents incremental innovation in a relatively loose domain – the robotics industry -- rather than in a tight domain (e.g., the finance industry). With the premise that people in all cultures have the same potential to innovate, it is conceivable that in a tight nation or province where many domains are under strict control, people tend to identify more opportunities and freedom in a loose domain and unleash their creativity there, more so than those who live in a loose country or province where many domains are already loose.

Accordingly, we predict a higher level of robot application in tight than in loose countries (Hypothesis 1). Following the same logic, within the same country where cultural tightness varies at the state/province level [12,36], we predict that tighter states/provinces will have higher robot application (Hypothesis 2). Finally, we apply the same logic to predict how country-level and state-/province-level tightness interact to influence robot application in the robotics industry (a loose domain). That is, within a tight country, when a state/province is also tight, a loose domain will energize people to be more active in searching for growth opportunities and engaging in innovative activities than when a state/province is loose, where there are more loose domains for people to unleash creativity. Meanwhile, within a loose country, while a loose domain in a tight state/ province offers people more freedom to exercise creativity in comparison to a loose state/province, the difference between a tight and a loose state/province will not be as pronounced as that in a tight country. We therefore predict that in a tight country such as China, the differences in robot application between a tight and a loose state/province will be significantly greater than those in a loose country such as the U.S. (Hypothesis 3).

## The current study

In light of the substantial societal implications associated with industrial robots [2], and in alignment with prior research [40,41], we operationalize robot application using two indices: industrial robot density and robot growth. Robot density refers to the operational stock of industrial robots per 10,000 labor force, and robot growth refers to the operational stock growth of industrial robots over the last year per 10,000 labor force.

We test our hypotheses with three studies using multi-source longitudinal archival databases (see S1 Table). We conducted three sets of Hierarchical Linear Modeling (HLM) regression analyses [3,42] to test the three hypotheses, respectively. To exclude the influence of time-series trends, we controlled for the year-fixed effects by coding each of the years as dummy variables in all models. Specifically, in Study 1, we analyzed robotics data across 32 countries and territories from IFR to test whether robot application is more prevalent in tight than in loose cultures (Hypothesis 1). In Study 2, we computed robotics data across 50 states in the United States and 31 provinces in China reported by IFR, the U.S. Bureau of Economic Analysis, and the National Bureau of Statistics of China, to examine the relationship between cultural tightness and robot application at the state or province level within each country. In Study 3, we tested Hypothesis 3 using both country and state/province-level data to examine the interaction effect of cultural tightness at the country level and the state/province level on robot applications within each state/province. Data and codes are available from https://osf.io/n6vuk/.

## Study 1: Cultural tightness and robot application across 32 countries and territories

### Measures

#### Cultural tightness.

We used country-level cultural tightness scores calculated by Gelfand et al. [12]. We averaged the cultural tightness scores of Germany (former East) and Germany (former West) as the cultural tightness scores of Germany. Thus, the final sample comprised 32 countries and territories. Higher scores of cultural tightness indicate a tighter culture.

#### Robot application.

We purchased robot data from IFR, which provides an operational stock of industrial robots across countries and territories from 1993 to 2022. Then we used the following two equations to calculate robot density and robot growth: For yearly robot density, we divided the operational stock of industrial robots by per 10,000 labor force (collected from the World Bank), as shown in Equation 1. We used Equation 2 to compute yearly robot growth.


$$Robot{\text{density}}_{it}=\frac{Robot{\text{stock}}_{it}}{Labor_{it}}$$ Equation 1

$$Robot{\text{growth}}_{it}=\frac{Robot{\text{stock}}_{it}-Robot{\text{stock}}_{it-1}}{Labor_{it}}$$ Equation 2

In equation 1, where $Robot{\text{density}}_{it}$ represents the density of industrial robots in region *i* and year *t*, $Robotstock_{it}$ represents the operational stocks of industrial robots in region *i* and year *t*, $Labor_{it}$ represents the number of labor force in region *i* and year *t*. In equation 2, $Robot{\text{growth}}_{it}$ represents the growth of industrial robots in region *i* and year *t*, and $Robot{\text{stock}}_{it-1}$ represents the operational stocks of industrial robots in region *i* and year *t-1*.

#### Control variables.

We collected each country-year GDP per capita based on purchasing power parity (PPP) in current U.S. dollars and unemployment rate from the World Bank. We log-transformed GDP per capita prior to analyses because it showed a strong positive skew. The unemployment rate refers to the share of the labor force that is without work but available for and seeking employment. Collectivism data was collected from Hofstede [43]. In line with previous literature [44], we reverse-coded Hofstede’s individualism index to denote collectivism (= 100 – individualism) for ease of interpretation.

All variables and their corresponding data sources are shown in S1 Table. Descriptive statistics of main variables in Study 1 are shown in S2 Table.

### Results

We predicted that in a loose domain like the robotics industry, cultural tightness would be positively related to robot application, operationalized as robot density and robot growth. We conducted regression analyses a) without any control variables, b) controlling for GDP per capita, and c) controlling for GDP per capita, unemployment rate, and collectivism. We controlled for GDP per capita because many of our focal variables are commonly associated with national wealth [12]. To isolate the effect of other economic-related variables on robot application across various countries, we controlled for the unemployment rate [2]. In addition, we controlled for collectivism as research showed its positive relationship with cultural tightness[12,45].

In support of Hypothesis 1, Table 1 shows that cultural tightness at the country level was positively associated with robot density across the globe (*b* = 0.25, *SE* = 0.16, *p =* 0.08). This positive association replicated and remained significant after controlling for GDP per capita (*b* = 0.28, *SE* = 0.13, *p =* 0.03), and also after controlling for the unemployment rate and collectivism (*b* = 0.28, *SE* = 0.15, *p =* 0.06). Moreover, we later controlled for the other four dimensions of Hofstede’ culture (i.e., power distance, masculinity, uncertainty avoidance and long-term orientation) [43], and the results remained the same (see S3B Table).

**Table 1:** Cultural tightness and robot application at the country level in Study 1.

Variables	Robot density			Robot growth		
	Model 1	Model 2	Model 3	Model 4	Model 5	Model 6
**Cultural tightness**	0.25^†^(0.14)	0.28*(0.13)	0.28^†^(0.15)	0.19^†^(0.10)	0.25*(0.10)	0.17(0.12)
**GDP per capita (log)**		0.20**(0.07)	0.22**(0.08)		0.34***(0.08)	0.44***(0.10)
**Unemployment rate**			−0.03(0.03)			−0.06(0.04)
**Collectivism**			0.02(0.15)			0.27*(0.12)
**Constant**	−0.34*(0.16)	−0.25(0.16)	−0.26(0.16)	0.70***(0.17)	0.59***(0.17)	0.51**(0.17)
**Observations**	959	947	918	927	917	889
**Log Likelihood**	–755.13	–743.89	–729.62	–1066.42	–1049.44	–1023.75

Standard errors in parentheses; All estimates have been standardized via z-scoring for presentation;

† *p* < 0.10, ^*^ *p* < 0.05, ^**^ *p* < 0.01, ^***^ *p* < 0.001.

**Fig 2:**
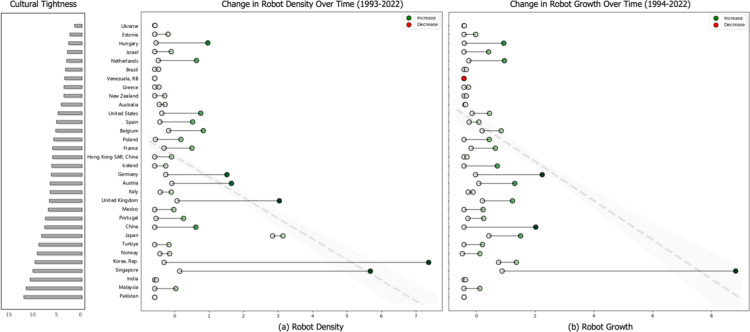
Cultural tightness and robot application across 32 countries and territories from 1993 to 2022 in Study 1. Each country is ordered in terms of cultural tightness. White and colored nodes represent each country’ a) robot density and b) robot growth, respectively. Greener nodes represent a greater extent of increase in a) robot density and b) robot growth, and red nodes represent declining a) robot density and b) robot growth. The dashed trend line represents the positive correlation between cultural tightness and a) robot density and b) robot growth, and the shaded region indicates standard error. The trendline shows that cultural tightness correlates positively with a) robot density and b) robot growth cross-sectionally, and the colored nodes show that countries with a tighter culture have a greater increase in a) robot density and b) robot growth than those with a looser culture. All estimates have been standardized via z-scoring.

Similar patterns were found for the relationship between cultural tightness and robot growth. Specifically, cultural tightness was positively related to robot growth (*b* = 0.19, *SE* = 0.10, *p =* 0.07), and this relationship remained significant after controlling for GDP per capita (*b* = 0.25, *SE* = 0.10, *p =* 0.01). However, it became nonsignificant when we controlled for the unemployment rate and collectivism (*b* = 0.17, *SE* = 0.12, *p* = 0.14). We speculate that this might be due to the potential conceptual overlap between cultural tightness and collectivism, as collectivism includes an element of norm compliance. Fig 2 visualizes these relationships.

These results provide overall support for Hypothesis 1, that is, in the nascent robotics industry (loose domain), people in tight countries seem to be more likely to embrace and engage in innovative activities to apply industrial robots in their work settings. Meanwhile, to further test our cross-level lens of tightness-looseness, we collected data from the financial industry (representing a tight domain) and found that people in tight countries were less likely to innovate, indicated by lower capital adequacy ratio (calculated from using the average Z score of the bank liquid reserves to bank assets ratio and bank capital to assets ratio between 1993 and 2022 from the World Bank). These results provide additional support for our theory. Please see Support information for details.

To check the robustness of our results, we conducted three additional analyses, detailed in the Supporting Information. First, we controlled for more variables, including education spending per GDP, R&D spending per GDP, and other four Hofstede’s culture dimensions, to isolate the effects of these factors on robot density and growth. All results remained the same (see S3B Table). Second, following Jackson et al.’s [3] approach, we tested whether cultural tightness interacted with time to predict robot application, and the key findings were replicated (see S3C, S3D and S3E Table). Finally, we collected data on robot installation density (i.e., installations of industrial robots per 10,000 labor force) for each country as an alternative outcome variable, and replicated the findings of all analyses (see S3F and S3G Table).

## Study 2: Cultural tightness and robot application within a country

### Measures

#### Cultural tightness.

We obtained cultural tightness across 50 United States from Harrington and Gelfand [13] and across 31 provinces in China from Chua et al. [19], respectively. Higher scores indicate tighter states/provinces.

#### Robot application.

Since IFR only presents industry data within countries and territories, it must be measured and matched to state-/province-level regions. Based on previous research [2,46], we conducted the Bartik IV method and calculated robot stock within a region with a formula shown in Equation 3.


$$Robot{\text{stock}}_{it}=\underset{j}{\sum }\frac{Labor_{ji}}{\sum_{j}Labor_{ji}}\times Robot_{jt}$$ Equation 3

Where $Labor_{ji}$ represents the number of labor force in region *i* and industry *j*, and $Robot_{jt}$ represents the number of industrial robots in industry *j* and year *t*.

For state-level robot stock in the U.S., we matched data from the U.S. Bureau of Economic Analysis and IFR by using seven main industries, including 1) primary industry, 2) mining and quarrying, 3) manufacturing, 4) electricity, gas, water supply (i.e., utilities), 5) construction, 6) education and R&D, and 7) all other non-manufacturing branches. To test the validity of our estimates, we combined our data with state-level robot data used by Yam et al. [41] in the U.S. The results showed that these two datasets were strongly correlated (for robot stock: *r* = 0.81, *p* < 0.001; for robot density: *r* = 0.83, *p* < 0.001), indicating that our estimates were robust.

For province-level robot stock in China, we matched data from the National Bureau of Statistics of China and IFR by employing the same seven main industries in the U.S.. We combined our data with province-level robot data provided by Du and Lin [40] to check the robustness of our estimates of robot data in China. The results indicated that the two datasets were highly correlated (for robot density: *r* = 0.88, *p* < 0.001; for robot installation density: *r* = 0.87, *p* < 0.001), suggesting our estimates were valid.

Then we followed Equations 1 and 2 in Study 1 to compute robot density and robot growth, respectively.

#### Control variables.

We controlled the same variables as we did in Study 1. For state-level control variables in the U.S., GDP per capita and unemployment rate were collected from the U.S. Bureau of Economic Analysis and Bureau of Labor Statistics, respectively. GDP per capita was positively skewed, so we log-transformed them prior to analyses. Collectivism was obtained from Vandello & Cohen’s index [47]. For province-level control variables in China, we collected GDP per capita and unemployment rate data from the National Bureau of Statistics of China. GDP per capita showed a skew and was log-transformed before estimation. Collectivism was obtained from Van de Vliert et al.’s index [48].

All variables and their corresponding data sources are presented in S1Table. Descriptive statistics of 50 U.S. states in Study 2a are presented in S4A Table and Descriptive statistics of 31 China provinces in Study 2b are presented in S4B Table, respectively.

### Results

In this study, we tested Hypothesis 2 using data from two distinct cultural contexts: the United States, characterized by a relatively loose culture, and China, noted for its tight culture [12]. As states/provinces within a country generally exhibit greater cultural homogeneity and show more similar degrees of technological development [13,19] than countries across the world, the investigation at the state/province level within a country provides a more rigorous test of our hypothesis.

Following Study 1, we used the same method to test the relationship between cultural tightness and robot application (i.e., robot density and robot growth) across 50 states in the U.S. (Study 2a) and 31 provinces in China (Study 2b), respectively.

**Fig 3:**
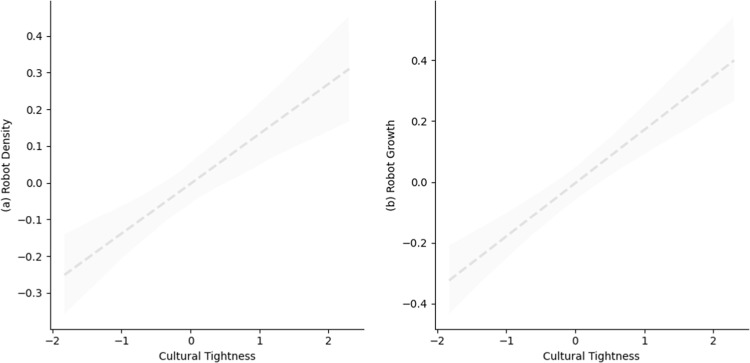
Correlation between cultural tightness and robot application across 50 states in the U.S. in Study 2a. The dashed trend line represents the positive correlation between cultural tightness and a) robot density and b) robot growth, respectively. The shaded region indicates standard error. All estimates have been standardized via z-scoring.

As shown in Table 2, in the U.S., a loose country, state tightness is positively related to both robot density (*b* = 0.13, *SE* = 0.05, *p* = 0.008) and robot growth (*b* = 0.18, *SE* = 0.07, *p* = 0.007). These positive associations remain significant after controlling GDP per capita (for robot density: *b* = 0.11, *SE* = 0.05, *p* = 0.045; for robot growth: *b* = 0.12, *SE* = 0.07, *p* = 0.07), as well as controlling unemployment rate and collectivism (for robot density: *b* = 0.12, *SE* = 0.05, *p* = 0.013; for robot growth: *b* = 0.14, *SE* = 0.06, *p* = 0.03). Fig 3 and S5A Fig display these results, respectively.

**Table 2:** Cultural tightness and robot application at the U.S. state level in Study 2a.

Variables	Robot density			Robot growth		
	Model 1	Model 2	Model 3	Model 4	Model 5	Model 6
**Cultural tightness**	0.13**(0.05)	0.11*(0.05)	0.12*(0.05)	0.18**(0.07)	0.12^†^(0.07)	0.14*(0.06)
**GDP per capita (log)**		−0.09*(0.04)	−0.09*(0.04)		−0.18***(0.06)	−0.17**(0.06)
**Unemployment rate**			−0.04*(0.02)			−0.06**(0.02)
**Collectivism**			−0.09^†^(0.05)			−0.10(0.06)
**Constant**	−1.03***(0.07)	−1.16***(0.09)	−1.19***(0.09)	0.41***(0.09)	0.69***(0.12)	0.61***(0.12)
**Observations**	1188	1188	1178	1115	1115	1107
**Log Likelihood**	−476.60	−474.61	−454.24	−646.51	−641.06	−629.67

Standard errors in parentheses; All estimates have been standardized via z-scoring for presentation;

† *p* < 0.10, ^*^ *p* < 0.05, ^**^ *p* < 0.01, ^***^ *p* < 0.001.

Results were replicated when we further controlled for education spending per GDP and R&D spending per GDP (see S6A Table), and interacted cultural tightness with year within the U.S. (see S6C Table). We also calculated state-level robot installation density as an alternative outcome variable and were able to replicate all results (see S6E Table).

**Fig 4:**
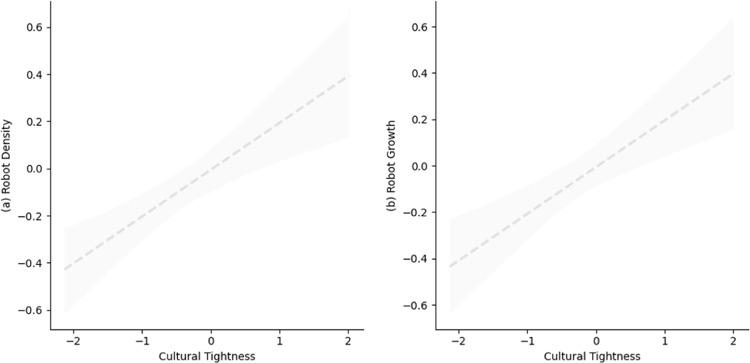
Correlation between cultural tightness and robot application across 31 Provinces in China in Study 2b. The dashed trend line represents the positive correlation between cultural tightness and a) robot density and b) robot growth, respectively. The shaded region indicates standard error. All estimates have been standardized via z-scoring.

In China, a tight country, results (see Table 3) indicate that province tightness is significantly related to both robot density (*b* = 0.20, *SE* = 0.06, *p* < 0.001) and robot growth (*b* = 0.20, *SE* = 0.07, *p* = 0.002). Moreover, these positive relationships remain significant when we controlled GDP per capita (for robot density: *b* = 0.27, *SE* = 0.07, *p* < 0.001; for robot growth: *b* = 0.19, *SE* = 0.08, *p* = 0.02), and unemployment rate and collectivism (for robot density: *b* = 0.23, *SE* = 0.06, *p* < 0.001; for robot growth: *b* = 0.21, *SE* = 0.07, *p* = 0.002). Fig 4 and S5B Fig depict these results, respectively.

**Table 3:** Cultural tightness and robot application at the China province level in Study 2b.

Variables	Robot density			Robot growth		
	Model 1	Model 2	Model 3	Model 4	Model 5	Model 6
**Cultural tightness**	0.20***(0.06)	0.27***(0.07)	0.23***(0.06)	0.20**(0.07)	0.19*(0.08)	0.21**(0.07)
**GDP per capita (log)**		−0.13(0.09)	−0.12(0.07)		0.03(0.10)	−0.05(0.10)
**Unemployment rate**			0.02(0.03)			−0.01(0.03)
**Collectivism**			−0.09^†^(0.05)			−0.12*(0.06)
**Constant**	−0.83***(0.09)	−1.00***(0.14)	−0.98***(0.12)	1.82***(0.10)	1.78***(0.14)	1.50***(0.12)
**Observations**	453	453	422	420	420	389
**Log Likelihood**	−239.38	−238.43	−138.23	−277.20	−277.14	−216.70

Standard errors in parentheses; All estimates have been standardized via z-scoring for presentation;

† *p* < 0.10, ^*^ *p* < 0.05, ^**^ *p* < 0.01, ^***^ *p* < 0.001.

The above findings were also held when we further controlled for education spending per GDP and R&D spending per GDP (see S6B Table), and interacted cultural tightness with year within China (see S6D Table). We also estimated province-level robot installation density as an alternative outcome variable and were able to replicate our results (see S6F Table).

In sum, the results reported above provide considerable support for Hypothesis 2, which states that cultural tightness at the state/province level is positively related to robot application, an incremental innovation in a loose domain. Study 2 provides strong evidence replicating the key findings in Study 1, that is, in a loose domain such as the robotics industry, cultural tightness is positively related to incremental innovation.

## Study 3: Cross-level interaction effects of cultural tightness on robot application

### Measures

All data used in Study 3 were sourced from Study 2.

### Results

We integrated the data from Study 2 to test Hypothesis 3 regarding the cross-level interaction effect between country- and state/province-level cultural tightness on robot density and robot growth. The available data for the U.S. is from 1998–2022, but for China is from 2008–2022. We conducted a series of regression analyses a) without any control variables, b) controlling for the interaction of state-/province-level cultural tightness with year and GDP per capita, and c) further controlling for unemployment rate and collectivism. We accounted for the interaction between state-/province-level cultural tightness and year in our analysis to address variations in the temporal development of robot application between the United States and China [2,46]. In addition, our additional analyses in Studies 1 and 2 also revealed that the interaction term of cultural tightness × year was positively related to robot application. All variables used in Study 3 were z-scored by country before estimation.

For robot density, the interaction between country and state-/province-level cultural tightness was positive but not significant (*b* = 0.03, *SE* = 0.03, *p =* 0.37) without any control variables. However, this interaction became significant after controlling the interaction of state-/province-level cultural tightness with year and GDP per capita (*b* = 0.10, *SE* = 0.04, *p =* 0.008), and remained significant after further controlling the unemployment rate and collectivism (*b* = 0.06, *SE* = 0.03, *p =* 0.098).

**Fig 5:**
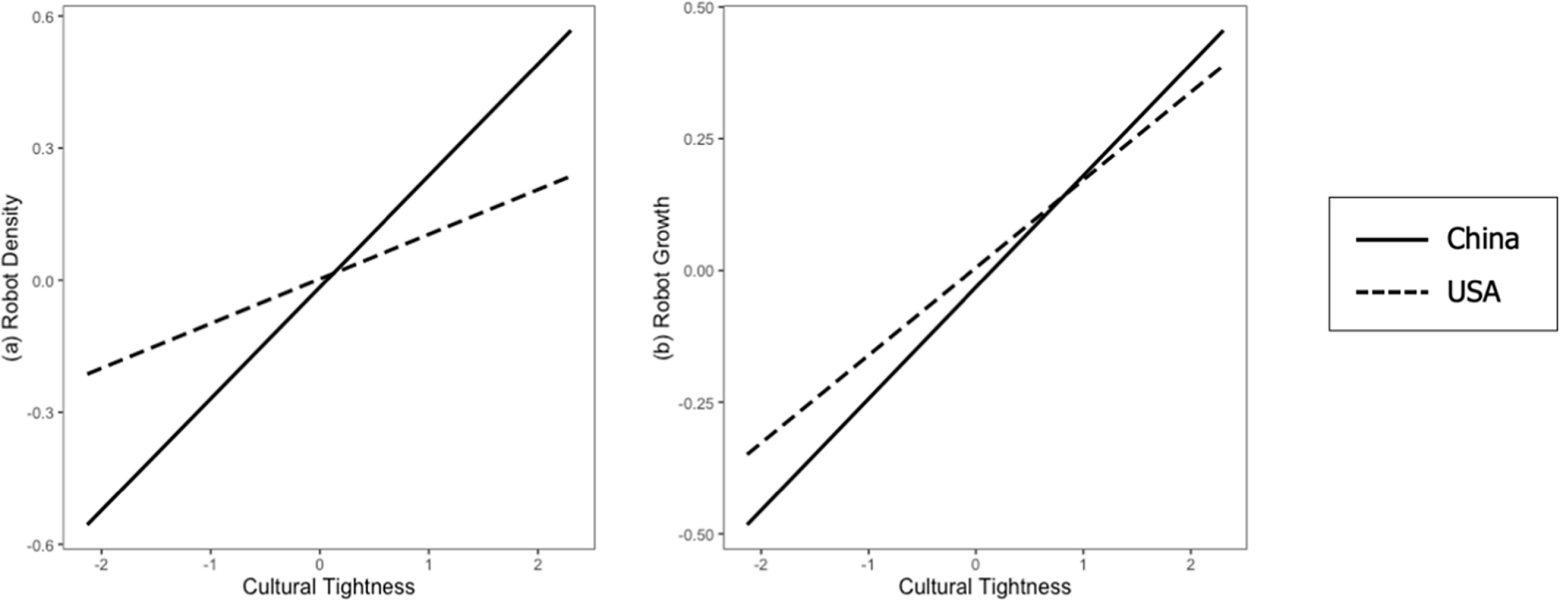
Interaction effect of country- and state-/province-level cultural tightness on robot application in Study 3. The solid trend lines represent the positive correlations between cultural tightness and a) robot density and b) robot growth across 31 provinces in China. The dashed trend lines represent the positive correlations between cultural tightness and a) robot density and b) robot growth across 50 states in the U.S. All estimates have been standardized via z-scoring.

For robot growth, similar patterns appeared. The interaction effect of state-/province-level × cultural level cultural tightness (U.S. vs. China) was not significant (*b* = 0.01, *SE* = 0.04, *p =* 0.84). However, this effect became significant when we controlled the interaction of state-/province-level cultural tightness with year and GDP per capita (*b* = 0.10, *SE* = 0.05, *p =* 0.03), and remained significant when we further controlled unemployment rate and collectivism (*b* = 0.07, *SE* = 0.05, *p =* 0.099). These results are displayed in Table 4 and Fig 5.

**Table 4:** The interaction effect of country- and state-/province-level cultural tightness on robot application in Study 3.

Variables	Robot density			Robot growth		
	Model 1	Model 2	Model 3	Model 4	Model 5	Model 6
**State-/Province-level cultural tightness**	0.15***(0.04)	0.13**(0.04)	0.15***(0.04)	0.18***(0.05)	0.15**(0.05)	0.16***(0.05)
**Country**	−0.28***(0.04)	−0.12**(0.04)	−0.26***(0.04)	−0.17***(0.04)	0.39***(0.05)	0.32***(0.05)
**State-/Province-level cultural tightness × Country**	0.03(0.03)	0.10**(0.04)	0.06^†^(0.03)	0.01(0.04)	0.10*(0.05)	0.07^†^(0.05)
**Year**		0.66***(0.06)	0.29***(0.06)		2.14***(0.08)	1.98***(0.10)
**State-/Province-level cultural tightness × Year**		0.13***(0.01)	0.12***(0.01)		0.09***(0.01)	0.09***(0.01)
**GDP per capita (log)**		−0.20***(0.04)	−0.13***(0.04)		−0.27***(0.05)	−0.23***(0.05)
**Unemployment rate**			−0.03*(0.01)			−0.07***(0.02)
**Collectivism**			−0.09*(0.04)			−0.12*(0.05)
**Constant**	−1.20***(0.07)	−0.30*(0.12)	−0.90***(0.12)	1.05***(0.08)	−2.31***(0.16)	−2.43***(0.16)
**Observations**	1641	1641	1600	1535	1535	1496
**Log Likelihood**	−917.12	−759.96	−578.71	−1419.58	−1131.84	−1055.32

Country: 0 = U.S., 1 = China; Standard errors in parentheses; All estimates have been standardized via z-scoring for presentation;

† *p* < 0.10, ^*^ *p* < 0.05, ^**^ *p* < 0.01, ^***^ *p* < 0.001.

We reckon that it may not be surprising that the interaction effect between state-/province-level and country-level cultural tightness on robot application was not significant without any control variables. As the United States has been the biggest economy in the world for decades, while China is still a developing country with a population several times larger than that of the United States, the gap in economic size requires us to control GDP per capita. In addition, according to IFR, China has applied robots rapidly in the past decade – as in 2006, it had less than 20,000 robots [46], during which time the United States already had more than 150,000 robots [2] – with an average growth of more than 13,000 robots every year from 2006 to 2022. The temporal dimension of robot application between these two countries thus requires the control for the interaction of state-/province-level cultural tightness by year. In other words, the real test of Hypothesis 3 demands the controlling of these two variables.

Therefore, we conclude that our results support Hypothesis 3. As shown in Fig 5, the simple slope analysis shows a greater difference in robot application between tight and loose provinces in China than between tight and loose states in the U.S.

## Discussions

This paper proposes and tests a cross-level lens of cultural tightness-looseness to explain the disparity in industrial robot application across countries/territories in the world and across states/provinces within a country. Findings from three studies using multi-source longitudinal archival data provided consistent support for our central thesis that the first or second-level cultural tightness is positively related to incremental innovation in the third-level loose domain, represented in this study by the robotics industry. Specifically, Study 1 showed that cultural tightness was positively related to robot application using data from 32 countries and territories in the past 30 years, and such a positive relationship was robust after a host of control variables at the country level was accounted for. Study 2 showed that across 50 states in the U.S., robot density and growth were higher in tighter states, and the same phenomenon was observed across 31 provinces in China, where the tighter provinces had higher robot density and growth. These relationships held when we controlled a host of variables at the state/province level. More importantly, Study 3 demonstrated an interactive effect between country-level and state-/province-level cultural tightness on robot application, which showed a stronger association between state/province tightness and robot application within a tight country (China) than within a loose one (the U.S.).

### Contributions

Our findings make important theoretical contributions to the literature on cultural tightness-looseness and organization innovation manifested in robot applications.

1. We significantly extend the theory of cultural tightness from a uni-level construct [13] to include the second- and third-level domain tightness to capture the cross-level interaction effect, making more precise predictions about incremental innovation in a loose domain [21]. The addition of domain tightness not only explains earlier seemingly counterintuitive findings [19] but also predicts the new phenomena of robot application disparity across countries, as well as disparity across states/provinces within a country. The cross-level lens of cultural tightness-looseness underscores the significance of recognizing and distinguishing the domanial strength (i.e., loose vs. tight domain) to account for incremental innovation in a specific domain [38]. Our research offers novel theoretical insights into the understanding of how cultural tightness functions at multiple levels to influence innovative behaviors, which has been overlooked by previous research that predominantly investigated cultural tightness at a single level to predict its effects.
2. Our findings contribute to the cultural psychology literature by challenging the basic assumption that cultural tightness is always positively related to restricted behaviors and negatively related to innovation [23]. Instead, we provide a more nuanced picture regarding the relationship between cultural tightness and innovation [12,13,16,19]. Our findings suggest that it will depend on domain tightness. While in a tight domain like the finance industry, people in tight cultures are less innovative than those in a loose culture, in loose domains such as the robotics industry, people in tighter cultures are in fact more active in engaging in innovative activities [49], resulting in higher levels of robot application, both across and within countries.
3. Our findings offer new insights into the complexity involved in explaining how cultural tightness might be related to incremental innovation. It seems that lower-level domain tightness, rather than higher-level cultural tightness, is a more proximate predictor of robot application. That is, domain tightness is a direct predictor of people’s engagement in innovative activities: the looser is a domain, the higher is the innovative activity. And this relationship is amplified by higher-level cultural tightness. These findings indirectly support our premise that people in all cultures have the impetus to innovate, as long as they are given the opportunity and permission.

A caution is warranted regarding the practical implications of our findings, however. To be clear, we do not advocate increasing tightness at the cultural level to promote innovation at the domain level, because ultimately, it’s the looseness (or freedom) that fosters creativity. Furthermore, in a tight culture, there would be little innovation in many other domains, which would be detrimental to the large innovation landscape in that culture. More seriously, when people can find only limited loose domains to exercise their creativity, it could lead to a crowding effect that increases involution within these limited domains.

### Methodological strengths, limitations and future directions

There are several notable methodological strengths in this research. First, we obtained objective industrial robot data from credible multi-source archive databases such as IFR, the U.S. Bureau of Economic Analysis, and the National Bureau of Statistics of China. We also triangulated our data with those used in other studies [39,40] to ensure accuracy. Second, we conducted rigorous analyses to test each hypothesis first without any control variables, then gradually adding variables that either have been shown to affect robot application or may potentially influence robot application, to make sure that our findings are robust and stable. Lastly, we adopted a stepwise approach to carefully test our proposition that cultural tightness is positively related to incremental innovation in a loose domain. Namely, we first tested it at the country level, then at the state/province level, and finally, country-level and state/province level interaction. This step-wise approach increases our confidence along the way about the predictive power of the cross-level lens of cultural tightness-looseness.

While this study offers compelling evidence about how cultural tightness-looseness at different levels interact to explain the large disparity in robot application, we encourage future research to explore additional perspectives (e.g., institutional, sociological, economic, technological, psychological) that may elucidate why and how certain countries and regions have higher robot density and faster growth of robot application than others [7]. Furthermore, it will be desirable to test the generalizability and reliability of our findings by using data from other tight and loose cultures (e.g., Thailand, Indonesia, Canada, and Australia).

Going beyond robot application, future studies can also test the cross-level lens of cultural tightness in other tight and loose industries (e.g., education and new energy) to examine its predictive power. For example, we have tested our theory by using data in a financial industry (a tight domain) and found support for this theory (see Table S3A in Supporting Information). Furthermore, if we define the first-level cultural tightness at the organizational level, then functional departments embedded in the organization can be viewed as the second-level domain, and our cross-level lens of cultural tightness can then be tested by sampling a large number of organizations with different cultural tightness, then measuring the domain tightness of all departments (e.g., R&D, HR, Marketing, Sales, Production) within each organization and the innovation output of each department [38]. It is evident that the cross-level lens of cultural tightness has flexibility in predicting innovation in specific situated contexts.

## Conclusion

This study provides considerable support for our cross-level lens of cultural tightness in explaining and predicting the disparity in robot application across countries and regions. It shows that lower-level domain looseness is the key driver of incremental innovation, which is amplified by higher-level cultural tightness. Our findings significantly challenge and extend the existing uni-level theory of cultural tightness, revealing how cultural tightness functions at multiple levels to influence incremental innovation (e.g., robot application) in a loose industry (e.g., the robotics industry). Additionally, our findings seem to support the general premise that people in all cultures have the impetus to innovate if they are given the freedom and opportunity.

## Supporting information

S1 Table Variable names and data sources. (DOCX)

S2 Table Descriptive statistics of main variables in Study 1. (DOCX)

S3 Table Additional analyses for Study 1. **S3A Table.** Cultural tightness and capital adequacy ratio at the country level in Study 1. **S3B Table.** Cultural tightness and robot application at the country level in Study 1. **S3C Table.** The interaction effect of cultural tightness and year on robot density at the country level in Study 1. **S3D Table.** The interaction effect of cultural tightness and year on robot growth at country level in Study 1. **S3E Table.** The interaction effect of cultural tightness and year on capital adequacy ratio at the country level in Study 1. **S3F Table.** Cultural tightness and robot installation density at the country level in Study 1. **S3G Table**. The interaction effect of cultural tightness and year on robot installation density at the country level in Study 1.
(DOCX)

S4 Table Descriptive statistics of main variables in Study 2. **S4A Table.** Descriptive statistics of 50 U.S. states in Study 2a. **S4B Table.** Descriptive statistics of 31 China provinces in Study 2b.
(DOCX)

S5 Fig Additional figs for Study 2. **S5A Fig.** Culture tightness and robot application across 50 U.S. states from 1998 to 2022 in Study 2a. **S5B Fig.** Culture tightness and robot application across 31 provinces in China from 2008 to 2022 in Study 2b.
(DOCX)

S6 Table Additional analyses for Study 2. **S6A Table.** Cultural tightness and robot application at the U.S. state level in Study 2a. **S6B Table.** Cultural tightness and robot application at the China province level in Study 2b. **S6C Table.** The interaction effect of cultural tightness and year on robot application at the U.S. state level in Study 2a. **S6D Table.** The interaction effect of cultural tightness and year on robot application at the China province level in Study 2b. **S6E Table.** Cultural tightness and robot installation density at the U.S. state level in Study 2a. **S6F Table.** Cultural tightness and robot installation density at the China province level in Study 2b.
(DOCX)
