# Supplementary material for: Explaining disparities in robot applications among nations and regions: A cross-level lens of cultural tightness-looseness
Source: PLoS One. 2025 Apr 16;20(4):e0321173. doi: 10.1371/journal.pone.0321173 (PMC12002431; doi:10.1371/journal.pone.0321173)
Supplement: S1 Table — (DOCX) [file pone.0321173.s001.docx]

**S1 Table. Variable names and data sources.**

| **Variables** | **Level** | **Year** | **Source** |
| --- | --- | --- | --- |
| **Cultural tightness across 32 countries and territories** | Country |  | Gelfand et al. (2011) [1] |
| **Cultural tightness across 50 states** | U.S. state |  | Harrington & Gelfand (2014) [2] |
| **Cultural tightness across 31 provinces** | China province |  | Chua et al. (2019) [3] |
| **Robot density** | Country | 1993–2022 | Operational stock of industrial robots: <https://ifr.org/worldrobotics/>  Labor force: <https://databank.worldbank.org/source/world-development-indicators> |
| **Robot growth** | Country | 1994–2022 |  |
| **Robot density** | U.S. state | 1998–2022 | Operational stock of industrial robots: <https://ifr.org/worldrobotics/>  Labor force by industry: <https://www.bea.gov/> |
| **Robot growth** | U.S. state | 1999–2022 |  |
| **Robot density** | China province | 2008–2022 | Operational stock of industrial robots: <https://ifr.org/worldrobotics/>  Labor force by industry: <https://data.stats.gov.cn> |
| **Robot growth** | China province | 2009–2022 |  |
| **GDP per capita** | Country | 1993–2022 | <https://databank.worldbank.org/source/world-development-indicators> |
| **GDP per capita** | U.S. state | 1998–2022 | <https://www.bea.gov/data/gdp/gdp-state> |
| **GDP per capita** | China province | 2008–2022 | <https://data.stats.gov.cn> |
| **Unemployment rate** | Country | 1993–2022 | <https://databank.worldbank.org/source/world-development-indicators> |
| **Unemployment rate** | U.S. state | 1998–2022 | <https://www.bls.gov/web/laus/laumstrk.htm> |
| **Unemployment rate** | China province | 2008–2021 | <https://data.stats.gov.cn> |
| **Collectivism** | Country |  | Hofstede (2001) [4] |
| **Collectivism** | U.S. state |  | Vandello & Cohen (1999) [5] |
| **Collectivism** | China province |  | Van de Vliert et al. (2013) [6] |

**References**

1. Gelfand MJ, Raver JL, Nishii L, Leslie LM, Lun J, Lim BC, et al. Differences Between Tight and Loose Cultures: A 33-Nation Study. Science. 2011;332: 1100–1104. doi:10.1126/science.1197754

2. Harrington JR, Gelfand MJ. Tightness–looseness across the 50 united states. Proc Natl Acad Sci USA. 2014;111: 7990–7995. doi:10.1073/pnas.1317937111

3. Chua RYJ, Huang KG, Jin M. Mapping cultural tightness and its links to innovation, urbanization, and happiness across 31 provinces in China. Proc Natl Acad Sci USA. 2019;116: 6720–6725. doi:10.1073/pnas.1815723116

4. Hofstede G. Culture’s consequences: Comparing values, behaviors, institutions and organizations across nations. Sage; 2001.

5. Vandello JA, Cohen D. Patterns of individualism and collectivism across the United States. Journal of Personality and Social Psychology. 1999;77: 279–292. doi:10.1037/0022-3514.77.2.279

6. Van De Vliert E, Yang H, Wang Y, Ren X. Climato-Economic Imprints on Chinese Collectivism. Journal of Cross-Cultural Psychology. 2013;44: 589–605. doi:10.1177/0022022112463605
