# Supplementary material for: Explaining disparities in robot applications among nations and regions: A cross-level lens of cultural tightness-looseness
Source: PLoS One. 2025 Apr 16;20(4):e0321173. doi: 10.1371/journal.pone.0321173 (PMC12002431; doi:10.1371/journal.pone.0321173)
Supplement: S2 Table — (DOCX) [file pone.0321173.s002.docx]

**S2 Table. Descriptive statistics of main variables in Study 1.**

| **Country/ Territory** | **Cultural tightness** | **Robot density** | | **Robot growth** | |
| --- | --- | --- | --- | --- | --- |
|  |  | **Mean** | **Years available** | **Mean** | **Years available** |
| **Australia** | 4.4 | 4.46 | 30 | 0.17 | 29 |
| **Austria** | 6.8 | 14.01 | 30 | 1.11 | 29 |
| **Belgium** | 5.6 | 14.14 | 30 | 0.69 | 29 |
| **Brazil** | 3.5 | 0.61 | 30 | 0.07 | 29 |
| **China** | 7.9 | 2.97 | 30 | 0.68 | 29 |
| **Estonia** | 2.6 | 1.15 | 30 | 0.22 | 29 |
| **France** | 6.3 | 9.98 | 30 | 0.52 | 29 |
| **Germany** | 7.0 | 33.17 | 30 | 1.80 | 29 |
| **Greece** | 3.9 | 0.54 | 30 | 0.06 | 29 |
| **Hong Kong SAR, China** | 6.3 | 2.36 | 30 | 0.26 | 29 |
| **Hungary** | 2.9 | 6.53 | 30 | 0.90 | 29 |
| **Iceland** | 6.4 | 0.87 | 30 | 0.19 | 29 |
| **India** | 11.0 | 0.17 | 30 | 0.03 | 29 |
| **Israel** | 3.1 | 1.91 | 30 | 0.30 | 29 |
| **Italy** | 6.8 | 21.61 | 30 | 1.03 | 29 |
| **Japan** | 8.6 | 52.47 | 30 | 0.22 | 29 |
| **Korea, Rep.** | 10.0 | 47.63 | 30 | 4.73 | 29 |
| **Malaysia** | 11.8 | 3.07 | 30 | 0.41 | 29 |
| **Mexico** | 7.2 | 1.77 | 30 | 0.33 | 29 |
| **Netherlands** | 3.3 | 7.18 | 30 | 0.69 | 29 |
| **New Zealand** | 3.9 | 1.71 | 30 | 0.19 | 29 |
| **Norway** | 9.5 | 3.53 | 30 | 0.20 | 29 |
| **Pakistan** | 12.3 | 0.00 | 30 | 0.00 | 29 |
| **Poland** | 6.0 | 2.86 | 30 | 0.42 | 29 |
| **Portugal** | 7.8 | 4.72 | 30 | 0.45 | 29 |
| **Singapore** | 10.4 | 30.81 | 30 | 3.57 | 29 |
| **Spain** | 5.4 | 10.59 | 30 | 0.65 | 29 |
| **Turkiye** | 9.2 | 1.51 | 30 | 0.25 | 29 |
| **Ukraine** | 1.6 | 0.03 | 29 | 0.00 | 28 |
| **United Kingdom** | 6.9 | 4.72 | 30 | 0.20 | 29 |
| **United States** | 5.1 | 10.67 | 30 | 0.71 | 29 |
| **Venezuela, RB** | 3.7 | 0.01 | 30 | 0.00 | 29 |
