# Supplementary material for: Explaining disparities in robot applications among nations and regions: A cross-level lens of cultural tightness-looseness
Source: PLoS One. 2025 Apr 16;20(4):e0321173. doi: 10.1371/journal.pone.0321173 (PMC12002431; doi:10.1371/journal.pone.0321173)
Supplement: S3 Table — S3A Table. Cultural tightness and capital adequacy ratio at the country level in Study 1. S3B Table. Cultural tightness and robot application at the country level in Study 1. S3C Table. The interaction effect of cultural tightness and year on robot density at the country level in Study 1. S3D Table. The interaction effect of cultural tightness and year on robot growth at country level in Study 1. S3E Table. The interaction effect of cultural tightness and year on capital adequacy ratio at the country level in Study 1. S3F Table. Cultural tightness and robot installation density at the country level in Study 1. S3G Table. The interaction effect of cultural tightness and year on robot installation density at the country level in Study 1. (DOCX) [file pone.0321173.s003.docx]

**S3 Table. Additional analyses for Study 1.**

**Cultural tightness and tight industry.** Capital adequacy ratio, a key index of financial regulation in the financial industry, refers to the extent to which assets are funded by other than own funds [1]. A high capital adequacy ratio indicates the strength of financial systems monitoring and shows the bank’s ability to provide funds to invest in innovative activity [2]. However, innovative activity inherently involves risk, and maintaining a high capital adequacy ratio often requires a conservative approach to risk, this conservative stance may be less inclined to allocate funds to potentially risky, innovative projects that could negatively affect their capital adequacy, which might inhibit investments in innovative activities [3,4].

We collected Bank liquid reserves to bank assets ratio and bank capital to assets ratio (1993–2022) from the World Bank. We averaged the z scores of these two indicators to get the composite score of capital adequacy ratio [1]. Then, we employed the same method as in Study 1 to test the relationship between cultural tightness and capital adequacy ratio across 32 countries and territories. As we predicted, S3A Table shows that in a tight domain, cultural tightness at the country level was negatively associated with the capital adequacy ratio across the globe after controlling for GDP per capita (*b* = –0.27, *SE* = 0.15, *p =* 0.07). This negative association replicated and remained significant after controlling for the unemployment rate and collectivism (*b* = –0.60, *SE* = 0.17, *p <* 0.001). Results were replicated when we further controlled for spending per GDP and R&D spending per GDP (*b* = –0.60, *SE* = 0.18, *p <* 0.001) and other four Hofstede’s culture dimensions (i.e., power distance, masculinity, uncertainty avoidance, and long-term orientation) [5] (*b* = –0.60, *SE* = 0.19, *p =* 0.001).

These results provide additional support for our theory, that’s, in a fully established industry (tight domain), people in tight countries are more tightly regulated, and not easy to acquire resources to innovate.

**S3A Table. Cultural tightness and capital adequacy ratio at the country level in Study 1.**

| **Variables** | **Model 1** | **Model 2** | **Model 3** | **Model 4** | **Model 5** |
| --- | --- | --- | --- | --- | --- |
| **Cultural tightness** | –0.14(0.15) | –0.27^†^(0.15) | –0.60^***^(0.17) | –0.60^***^(0.18) | –0.60^**^(0.19) |
| **GDP per capita (log)** |  | –0.73^***^(0.13) | –0.88^***^(0.14) | –0.62^***^(0.16) | –0.58^***^(0.16) |
| **Unemployment rate** |  |  | –0.08^†^(0.04) | –0.04(0.04) | –0.04(0.04) |
| **Collectivism** |  |  | 0.34^*^(0.17) | 0.34^†^(0.18) | 0.42^*^(0.20) |
| **Education spending per GDP** |  |  |  | –0.15^*^(0.06) | –0.15^*^(0.07) |
| **R&D spending per GDP** |  |  |  | –0.19^*^(0.09) | –0.17^†^(0.09) |
| **Power distance** |  |  |  |  | –0.05(0.20) |
| **Masculinity** |  |  |  |  | 0.12(0.15) |
| **Uncertainty avoidance** |  |  |  |  | –0.14(0.15) |
| **Long-term orientation** |  |  |  |  | –0.14(0.16) |
| **Constant** | 0.19(0.19) | 0.49^*^(0.19) | 0.56^**^(0.19) | –0.02(0.52) | –0.06(0.52) |
| **Observations** | 573 | 572 | 550 | 456 | 456 |
| **Log Likelihood** | –528.09 | –510.47 | –482.38 | –365.46 | –364.34 |

*Note.* Standard errors in parentheses; All estimates have been standardized via z-scoring for presentation; ^†^ *p* < 0.10, ^*^ *p* < 0.05, ^**^ *p* < 0.01, ^***^ *p* < 0.001.

We controlled for spending per GDP, R&D spending per GDP [6] and the other four Hofstede’s culture dimensions (i.e., power distance, masculinity, uncertainty avoidance, and long-term orientation) [5] to isolate the effect of these factors on our analyses across countries. Spending per GDP, R&D spending per GDP, energy development, and telecommunication development were collected from the World Bank. Country-level cultural data were collected from Hofstede [5]. All findings were replicated as shown in S3B Table.

**S3B Table. Cultural tightness and robot application at the country level in Study 1.**

| **Variables** | **Robot density** | | | **Robot growth** | | |
| --- | --- | --- | --- | --- | --- | --- |
|  | **Model 1** | **Model 2** | **Model 3** | **Model 4** | **Model 5** | **Model 6** |
| **Cultural tightness** | 0.28^*^(0.14) | 0.29^*^(0.14) | 0.30^**^(0.11) | 0.11(0.14) | 0.07(0.14) | 0.03(0.14) |
| **GDP per capita (log)** | 0.29^**^(0.09) | 0.58^***^(0.12) | 0.57^***^(0.11) | 0.42^**^(0.13) | 0.69^***^(0.18) | 0.67^***^(0.17) |
| **Unemployment rate** | 0.00(0.03) | 0.03(0.03) | 0.03(0.03) | –0.07(0.05) | –0.05(0.05) | –0.06(0.05) |
| **Collectivism** | 0.19(0.14) | 0.30^*^(0.15) | 0.13(0.12) | 0.30^*^(0.14) | 0.39^*^(0.15) | 0.31^*^(0.16) |
| **Education spending per GDP** | –0.06(0.04) | –0.02(0.04) | –0.01(0.04) | –0.16^*^(0.07) | –0.15^*^(0.08) | –0.13^†^(0.08) |
| **R&D spending per GDP** | 0.52^***^(0.06) | 0.49^***^(0.06) | 0.45^***^(0.06) | 0.19^*^(0.09) | 0.12(0.10) | 0.08(0.10) |
| **Energy development** |  | –0.12^**^(0.04) | –0.13^**^(0.04) |  | –0.20^**^(0.08) | –0.21^**^(0.08) |
| **Telecom. development** |  | –0.02(0.06) | –0.02(0.06) |  | –0.08(0.11) | –0.09(0.11) |
| **Power distance** |  |  | 0.19(0.12) |  |  | 0.07(0.15) |
| **Masculinity** |  |  | 0.19^*^(0.09) |  |  | –0.06(0.11) |
| **Uncertainty avoidance** |  |  | 0.22^*^(0.09) |  |  | 0.04(0.12) |
| **Long-term orientation** |  |  | 0.36^***^(0.09) |  |  | 0.24^*^(0.12) |
| **Constant** | 0.06(0.42) | 0.15(0.16) | 0.19(0.13) | –0.40(0.77) | 0.44^*^(0.21) | 0.49^*^(0.21) |
| **Observations** | 613 | 606 | 606 | 613 | 606 | 606 |
| **Log Likelihood** | –359.90 | –348.66 | –338.25 | –729.44 | –721.26 | –719.11 |

*Note.* Standard errors in parentheses; All estimates have been standardized via z-scoring for presentation; ^†^ *p* < 0.10, ^*^ *p* < 0.05, ^**^ *p* < 0.01, ^***^ *p* < 0.001.

Following Jackson et al.’s [7] approach, we conducted a longitudinal model with random intercepts and slopes across countries to test whether cultural tightness interacted with time to explain robot application. The interaction between country-level cultural tightness and year on both robot density (S3C Table, Model 1) and robot growth (S3D Table, Model 1) was positively significant. This effect was robust controlling for GDP per capita (S3C and S3D Table, Model 2), unemployment rate, and collectivism (S3C and S3D Table, Model 3). Results were replicated when we further controlled for spending per GDP and R&D spending per GDP (S3C and S3D Table, Model 4) and other four Hofstede’s culture dimensions (i.e., power distance, masculinity, uncertainty avoidance, and long-term orientation; Hofstede, 2001) [5] (S3C and S3D Table, Model 5). Findings were also held when we tested the interaction effect of cultural tightness and time on capital adequacy ratio (i.e., tight domain) (S3E Table). Therefore, we replicated all our findings by interacting country-level cultural tightness with year.

**S3C Table. The interaction effect of cultural tightness and year on robot density at the country level in Study 1.**

| **Variables** | **Model 1** | **Model 2** | **Model 3** | **Model 4** | **Model 5** |
| --- | --- | --- | --- | --- | --- |
| **Cultural tightness** | 0.25^†^(0.14) | 0.28^*^(0.13) | 0.26^†^(0.16) | 0.24^†^(0.14) | 0.27^*^(0.11) |
| **Year** | 0.32^***^(0.04) | 0.28^***^(0.04) | 0.27^***^(0.04) | –1.33(3.50) | –1.38(3.50) |
| **Cultural tightness × Year** | 0.10^***^(0.02) | 0.10^***^(0.02) | 0.10^***^(0.02) | 0.10^***^(0.02) | 0.10^***^(0.02) |
| **GDP per capita (log)** |  | 0.18^*^(0.07) | 0.16^*^(0.08) | 0.25^**^(0.09) | 0.28^***^(0.08) |
| **Unemployment rate** |  |  | –0.07^*^(0.03) | –0.02(0.03) | –0.02(0.03) |
| **Collectivism** |  |  | –0.00(0.15) | 0.19(0.14) | 0.04(0.12) |
| **Education spending per GDP** |  |  |  | –0.05(0.04) | –0.04(0.04) |
| **R&D spending per GDP** |  |  |  | 0.54^***^(0.06) | 0.50^***^(0.06) |
| **Power distance** |  |  |  |  | 0.16(0.11) |
| **Masculinity** |  |  |  |  | 0.16^†^(0.08) |
| **Uncertainty avoidance** |  |  |  |  | 0.21^*^(0.09) |
| **Long-term orientation** |  |  |  |  | 0.35^***^(0.09) |
| **Constant** | 0.19(0.15) | 0.22(0.14) | 0.20(0.15) | 2.29(5.48) | 2.39(5.48) |
| **Observations** | 959 | 947 | 918 | 613 | 613 |
| **Log Likelihood** | –733.36 | –725.60 | –713.72 | –349.77 | –339.73 |

*Note.* Standard errors in parentheses; All estimates have been standardized via z-scoring for presentation; ^†^ *p* < 0.10, ^*^ *p* < 0.05, ^**^ *p* < 0.01, ^***^ *p* < 0.001.

**S3D Table. The interaction effect of cultural tightness and year on robot growth at country level in Study 1.**

| **Variables** | **Model 1** | **Model 2** | **Model 3** | **Model 4** | **Model 5** |
| --- | --- | --- | --- | --- | --- |
| **Cultural tightness** | 0.18^†^(0.11) | 0.24^*^(0.10) | 0.14(0.12) | 0.03(0.13) | –0.01(0.13) |
| **Year** | –0.03(1.56) | –0.03(1.58) | –0.37(1.61) | –6.94(6.55) | –6.98(6.55) |
| **Cultural tightness × Year** | 0.14^***^(0.02) | 0.14^***^(0.02) | 0.14^***^(0.03) | 0.24^***^(0.04) | 0.24^***^(0.04) |
| **GDP per capita (log)** |  | 0.34^***^(0.08) | 0.39^***^(0.09) | 0.36^**^(0.13) | 0.34^**^(0.13) |
| **Unemployment rate** |  |  | –0.10^*^(0.04) | –0.10^*^(0.05) | –0.11^*^(0.05) |
| **Collectivism** |  |  | 0.25^*^(0.12) | 0.29^*^(0.14) | 0.21(0.14) |
| **Education spending per GDP** |  |  |  | –0.14^*^(0.07) | –0.13^†^(0.07) |
| **R&D spending per GDP** |  |  |  | 0.23^**^(0.09) | 0.21^*^(0.10) |
| **Power distance** |  |  |  |  | 0.09(0.14) |
| **Masculinity** |  |  |  |  | –0.09(0.10) |
| **Uncertainty avoidance** |  |  |  |  | 0.04(0.11) |
| **Long-term orientation** |  |  |  |  | 0.20^†^(0.11) |
| **Constant** | 0.73(2.52) | 0.62(2.55) | 1.11(2.60) | 11.25(10.25) | 11.34(10.25) |
| **Observations** | 927 | 917 | 889 | 613 | 613 |
| **Log Likelihood** | –1049.06 | –1033.82 | –1009.29 | –712.77 | –710.75 |

*Note.* Standard errors in parentheses; All estimates have been standardized via z-scoring for presentation; ^†^ *p* < 0.10, ^*^ *p* < 0.05, ^**^ *p* < 0.01, ^***^ *p* < 0.001.

**S3E Table. The interaction effect of cultural tightness and year on capital adequacy ratio at the country level in Study 1.**

| **Variables** | **Model 1** | **Model 2** | **Model 3** | **Model 4** | **Model 5** |
| --- | --- | --- | --- | --- | --- |
| **Cultural tightness** | 0.18^†^(0.11) | 0.24^*^(0.10) | 0.14(0.12) | 0.03(0.13) | –0.01(0.13) |
| **Year** | –0.03(1.56) | –0.03(1.58) | –0.37(1.61) | –6.94(6.55) | –6.98(6.55) |
| **Cultural tightness × Year** | 0.14^***^(0.02) | 0.14^***^(0.02) | 0.14^***^(0.03) | 0.24^***^(0.04) | 0.24^***^(0.04) |
| **GDP per capita (log)** |  | 0.34^***^(0.08) | 0.39^***^(0.09) | 0.36^**^(0.13) | 0.34^**^(0.13) |
| **Unemployment rate** |  |  | –0.10^*^(0.04) | –0.10^*^(0.05) | –0.11^*^(0.05) |
| **Collectivism** |  |  | 0.25^*^(0.12) | 0.29^*^(0.14) | 0.21(0.14) |
| **Education spending per GDP** |  |  |  | –0.14^*^(0.07) | –0.13^†^(0.07) |
| **R&D spending per GDP** |  |  |  | 0.23^**^(0.09) | 0.21^*^(0.10) |
| **Power distance** |  |  |  |  | 0.09(0.14) |
| **Masculinity** |  |  |  |  | –0.09(0.10) |
| **Uncertainty avoidance** |  |  |  |  | 0.04(0.11) |
| **Long-term orientation** |  |  |  |  | 0.20^†^(0.11) |
| **Constant** | 0.73(2.52) | 0.62(2.55) | 1.11(2.60) | 11.25(10.25) | 11.34(10.25) |
| **Observations** | 927 | 917 | 889 | 613 | 613 |
| **Log Likelihood** | –1049.06 | –1033.82 | –1009.29 | –712.77 | –710.75 |

*Note.* Standard errors in parentheses; All estimates have been standardized via z-scoring for presentation; ^†^ *p* < 0.10, ^*^ *p* < 0.05, ^**^ *p* < 0.01, ^***^ *p* < 0.001.

In addition, we also collected robot installation density (i.e., installations of industrial robots per 10,000 labor force) in each country as a potential outcome variable. We applied Equation 1 from the manuscript to calculate robot installation density using data on country-level installations of industrial robots provided by IFR from 1993 to 2022. These results replicated the main effects (S3F Table, Models 1–5) and the significant interaction between country-level cultural tightness and year (S3G Table, Models 1–5).

**S3F Table. Cultural tightness and robot installation density at the country level in Study 1.**

| **Variables** | **Model 1** | **Model 2** | **Model 3** | **Model 4** | **Model 5** |
| --- | --- | --- | --- | --- | --- |
| **Cultural tightness** | 0.27^*^(0.13) | 0.31^**^(0.12) | 0.29^*^(0.14) | 0.28^*^(0.13) | 0.29^**^(0.11) |
| **GDP per capita (log)** |  | 0.27^***^(0.08) | 0.32^***^(0.09) | 0.41^***^(0.11) | 0.41^***^(0.10) |
| **Unemployment rate** |  |  | –0.03(0.03) | –0.01(0.04) | –0.02(0.04) |
| **Collectivism** |  |  | 0.12(0.14) | 0.21(0.13) | 0.10(0.12) |
| **Education spending per GDP** |  |  |  | –0.16^**^(0.05) | –0.13^*^(0.05) |
| **R&D spending per GDP** |  |  |  | 0.35^***^(0.07) | 0.31^***^(0.07) |
| **Power distance** |  |  |  |  | 0.10(0.11) |
| **Masculinity** |  |  |  |  | 0.11(0.08) |
| **Uncertainty avoidance** |  |  |  |  | 0.18^*^(0.09) |
| **Long-term orientation** |  |  |  |  | 0.36^***^(0.09) |
| **Constant** | –0.32^*^(0.16) | –0.19(0.16) | –0.19(0.16) | –0.32(0.56) | –0.31(0.55) |
| **Observations** | 959 | 947 | 918 | 613 | 613 |
| **Log Likelihood** | –875.26 | –861.06 | –843.43 | –535.66 | –526.79 |

*Note.* Standard errors in parentheses; All estimates have been standardized via z-scoring for presentation; ^*^ *p* < 0.05, ^**^ *p* < 0.01, ^***^ *p* < 0.001.

**S3G Table. The interaction effect of cultural tightness and year on robot installation density at the country level in Study 1.**

| **Variables** | **Model 1** | **Model 2** | **Model 3** | **Model 4** | **Model 5** |
| --- | --- | --- | --- | --- | --- |
| **Cultural tightness** | 0.27^*^(0.13) | 0.31^**^(0.12) | 0.27^†^(0.14) | 0.20(0.13) | 0.22^*^(0.11) |
| **GDP per capita (log)** |  | 0.25^**^(0.08) | 0.25^**^(0.09) | 0.35^**^(0.11) | 0.36^***^(0.10) |
| **Unemployment rate** |  |  | –0.07^*^(0.03) | –0.04(0.04) | –0.05(0.03) |
| **Collectivism** |  |  | 0.09(0.14) | 0.20(0.13) | 0.08(0.11) |
| **Education spending per GDP** |  |  |  | –0.15^**^(0.05) | –0.12^*^(0.05) |
| **R&D spending per GDP** |  |  |  | 0.39^***^(0.07) | 0.35^***^(0.07) |
| **Power distance** |  |  |  |  | 0.12(0.11) |
| **Masculinity** |  |  |  |  | 0.11(0.08) |
| **Uncertainty avoidance** |  |  |  |  | 0.18^*^(0.09) |
| **Long-term orientation** |  |  |  |  | 0.35^***^(0.09) |
| **Year** | 0.29^***^(0.04) | 0.22^***^(0.05) | 0.21^***^(0.05) | –3.80(4.66) | –3.92(4.66) |
| **Cultural tightness × Year** | 0.14^***^(0.02) | 0.14^***^(0.02) | 0.14^***^(0.02) | 0.20^***^(0.03) | 0.20^***^(0.03) |
| **Constant** | 0.16(0.15) | 0.19(0.14) | 0.17(0.14) | 6.06(7.28) | 6.29(7.28) |
| **Observations** | 959 | 947 | 918 | 613 | 613 |
| **Log Likelihood** | –843.77 | –833.41 | –818.09 | –513.52 | –504.52 |

*Note.* Standard errors in parentheses; All estimates have been standardized via z-scoring for presentation; ^†^ *p* < 0.10, ^*^ *p* < 0.05, ^**^ *p* < 0.01, ^***^ *p* < 0.001.

**References**

1. Bank for International Settlements. International Convergence of Capital Measurement and Capital Standards: A Revised Framework. 2005. Available: https://www.bis.org/publ/bcbs118.pdf

2. Moyer SE. Capital adequacy ratio regulations and accounting choices in commercial banks. Journal of Accounting and Economics. 1990;13: 123–154. doi:10.1016/0165-4101(90)90027-2

3. Davis EP, Karim D, Noel D. The bank capital-competition-risk nexus – A global perspective. Journal of International Financial Markets, Institutions and Money. 2020;65: 101169. doi:10.1016/j.intfin.2019.101169

4. Laeven L, Levine R. Bank governance, regulation and risk taking. Journal of Financial Economics. 2009;93: 259–275. doi:10.1016/j.jfineco.2008.09.003

5. Hofstede G. Culture’s consequences: Comparing values, behaviors, institutions and organizations across nations. Sage; 2001.

6. Kokko A, Tingvall PG, Videnord J. The Growth Effects of R&D Spending in the EU: A Meta-Analysis. Economics. 2015;9: 20150040. doi:10.5018/economics-ejournal.ja.2015-40

7. Jackson JC, Yam KC, Tang PM, Sibley CG, Waytz A. Exposure to automation explains religious declines. Proc Natl Acad Sci USA. 2023;120: e2304748120. doi:10.1073/pnas.2304748120
