# Supplementary material for: Explaining disparities in robot applications among nations and regions: A cross-level lens of cultural tightness-looseness
Source: PLoS One. 2025 Apr 16;20(4):e0321173. doi: 10.1371/journal.pone.0321173 (PMC12002431; doi:10.1371/journal.pone.0321173)
Supplement: S4 Table — S4A Table. Descriptive statistics of 50 U.S. states in Study 2a. S4B Table. Descriptive statistics of 31 China provinces in Study 2b. (DOCX) [file pone.0321173.s004.docx]

**S4 Table. Descriptive statistics of main variables in Study 2.**

**S4A Table. Descriptive statistics of 50 U.S. states in Study 2a.**

| **State** | **Cultural tightness** | **Robot density** | | **Robot growth** | |
| --- | --- | --- | --- | --- | --- |
|  |  | **Mean** | **Years available** | **Mean** | **Years available** |
| **Alabama** | 75.45 | 10.05 | 24 | 1.18 | 22 |
| **Alaska** | 38.43 | 3.26 | 23 | 0.36 | 20 |
| **Arizona** | 47.56 | 4.65 | 25 | 0.57 | 24 |
| **Arkansas** | 75.03 | 9.52 | 25 | 1.03 | 24 |
| **California** | 27.37 | 5.82 | 25 | 0.65 | 24 |
| **Colorado** | 42.92 | 4.14 | 25 | 0.49 | 24 |
| **Connecticut** | 36.37 | 6.26 | 23 | 0.71 | 21 |
| **Delaware** | 51.02 | 5.11 | 7 | 0.65 | 3 |
| **Florida** | 49.28 | 3.24 | 25 | 0.41 | 24 |
| **Georgia** | 60.26 | 6.46 | 25 | 0.76 | 24 |
| **Hawaii** | 36.49 | 1.81 | 24 | 0.18 | 22 |
| **Idaho** | 45.50 | 6.66 | 25 | 0.82 | 24 |
| **Illinois** | 45.95 | 7.35 | 25 | 0.77 | 24 |
| **Indiana** | 54.57 | 11.46 | 23 | 1.39 | 21 |
| **Iowa** | 49.02 | 10.31 | 25 | 1.12 | 24 |
| **Kansas** | 60.36 | 8.49 | 25 | 0.92 | 24 |
| **Kentucky** | 63.91 | 9.38 | 25 | 1.05 | 24 |
| **Louisiana** | 65.88 | 5.26 | 25 | 0.55 | 24 |
| **Maine** | 34.00 | 7.24 | 18 | 0.82 | 15 |
| **Maryland** | 45.50 | 3.17 | 25 | 0.35 | 24 |
| **Massachusetts** | 35.12 | 6.03 | 18 | 0.57 | 15 |
| **Michigan** | 48.93 | 10.32 | 25 | 1.14 | 24 |
| **Minnesota** | 47.84 | 8.51 | 25 | 0.93 | 24 |
| **Mississippi** | 78.86 | 8.92 | 25 | 0.98 | 24 |
| **Missouri** | 59.60 | 7.14 | 25 | 0.80 | 24 |
| **Montana** | 46.11 | 3.40 | 25 | 0.40 | 24 |
| **Nebraska** | 49.65 | 7.37 | 25 | 0.83 | 24 |
| **Nevada** | 33.61 | 3.03 | 25 | 0.42 | 24 |
| **New Hampshire** | 36.97 | 7.99 | 25 | 0.86 | 24 |
| **New Jersey** | 39.48 | 4.68 | 25 | 0.51 | 24 |
| **New Mexico** | 45.43 | 3.00 | 25 | 0.32 | 24 |
| **New York** | 39.42 | 3.80 | 25 | 0.40 | 24 |
| **North Carolina** | 60.67 | 8.19 | 24 | 0.95 | 22 |
| **North Dakota** | 51.44 | 4.54 | 25 | 0.53 | 24 |
| **Ohio** | 52.30 | 9.65 | 25 | 1.03 | 24 |
| **Oklahoma** | 75.03 | 5.99 | 25 | 0.62 | 24 |
| **Oregon** | 30.07 | 7.79 | 25 | 0.87 | 24 |
| **Pennsylvania** | 52.75 | 7.46 | 25 | 0.79 | 24 |
| **Rhode Island** | 43.23 | 4.36 | 13 | 0.64 | 9 |
| **South Carolina** | 61.39 | 8.68 | 25 | 1.02 | 24 |
| **South Dakota** | 51.14 | 7.36 | 24 | 0.85 | 22 |
| **Tennessee** | 68.81 | 8.43 | 25 | 0.98 | 24 |
| **Texas** | 67.54 | 5.39 | 25 | 0.64 | 24 |
| **Utah** | 49.69 | 6.69 | 25 | 0.86 | 24 |
| **Vermont** | 37.23 | 7.82 | 24 | 0.83 | 22 |
| **Virginia** | 57.37 | 4.65 | 25 | 0.52 | 24 |
| **Washington** | 31.06 | 6.55 | 25 | 0.70 | 24 |
| **West Virginia** | 52.48 | 5.35 | 25 | 0.54 | 24 |
| **Wisconsin** | 46.91 | 11.01 | 23 | 1.31 | 21 |
| **Wyoming** | 51.94 | 2.55 | 20 | 0.34 | 16 |

**S4B Table. Descriptive statistics of 31 China provinces in Study 2b.**

| **Province** | **Cultural tightness** | **Robot density** | | **Robot growth** | |
| --- | --- | --- | --- | --- | --- |
|  |  | **Mean** | **Years available** | **Mean** | **Years available** |
| **安徽省/Anhui** | 2.54 | 21.31 | 15 | 6.03 | 14 |
| **北京市/Beijing** | 4.48 | 8.37 | 15 | 1.87 | 14 |
| **重庆市/Chongqing** | 2.21 | 17.66 | 15 | 4.17 | 14 |
| **福建省/Fujian** | 3.44 | 26.61 | 15 | 5.67 | 14 |
| **甘肃省/Gansu** | 2.01 | 10.92 | 15 | 2.75 | 14 |
| **广东省/Guangdong** | 5.00 | 37.44 | 15 | 9.16 | 14 |
| **广西壮族自治区/Guangxi** | 3.33 | 13.35 | 15 | 3.40 | 14 |
| **贵州省/Guizhou** | 0.85 | 9.78 | 15 | 2.54 | 14 |
| **海南省/Hainan** | 1.84 | 7.41 | 15 | 2.09 | 14 |
| **河北省/Hebei** | 3.60 | 16.82 | 15 | 4.04 | 14 |
| **河南省/Henan** | 3.04 | 21.47 | 15 | 4.26 | 14 |
| **黑龙江省/Heilongjiang** | 2.81 | 9.42 | 15 | 1.84 | 14 |
| **湖北省/Hubei** | 3.24 | 20.01 | 15 | 4.83 | 14 |
| **湖南省/Hunan** | 2.01 | 16.13 | 15 | 4.20 | 14 |
| **吉林省/Jilin** | 3.50 | 17.80 | 15 | 3.55 | 14 |
| **江苏省/Jiangsu** | 4.18 | 31.97 | 15 | 7.70 | 14 |
| **江西省/Jiangxi** | 2.61 | 22.33 | 15 | 5.34 | 14 |
| **辽宁省/Liaoning** | 3.99 | 20.23 | 15 | 4.40 | 14 |
| **内蒙古自治区/ Inner Mongolia** | 2.23 | 12.23 | 15 | 3.37 | 14 |
| **宁夏回族自治区/Ningxia** | 1.83 | 20.21 | 9 | 6.33 | 7 |
| **青海省/Qinghai** | 2.06 | 13.82 | 15 | 3.79 | 14 |
| **山东省/Shandong** | 4.25 | 24.90 | 15 | 5.69 | 14 |
| **山西省/Shanxi** | 1.95 | 12.60 | 15 | 3.18 | 14 |
| **陕西省/Shaanxi** | 2.62 | 15.95 | 15 | 3.92 | 14 |
| **上海市/Shanghai** | 4.87 | 20.41 | 15 | 4.68 | 14 |
| **四川省/Sichuan** | 3.11 | 15.87 | 15 | 4.31 | 14 |
| **天津市/Tianjin** | 2.70 | 25.03 | 15 | 5.66 | 14 |
| **西藏自治区/Tibet** | 3.20 | 5.48 | 9 | 1.52 | 7 |
| **新疆维吾尔自治区/Xinjiang** | 2.77 | 9.84 | 15 | 2.49 | 14 |
| **云南省/Yunnan** | 1.85 | 11.69 | 15 | 2.40 | 14 |
| **浙江省/Zhejiang** | 4.36 | 27.74 | 15 | 7.28 | 14 |
