# Supplementary material for: Explaining disparities in robot applications among nations and regions: A cross-level lens of cultural tightness-looseness
Source: PLoS One. 2025 Apr 16;20(4):e0321173. doi: 10.1371/journal.pone.0321173 (PMC12002431; doi:10.1371/journal.pone.0321173)
Supplement: S5 Fig — S5A Fig. Culture tightness and robot application across 50 U.S. states from 1998 to 2022 in Study 2a. S5B Fig. Culture tightness and robot application across 31 provinces in China from 2008 to 2022 in Study 2b. (DOCX) [file pone.0321173.s005.docx]

**S5 Fig. Additional figs for Study 2.**

We further visualized these results found in Study 2 as depicted in Figs S5A and S5B.

**
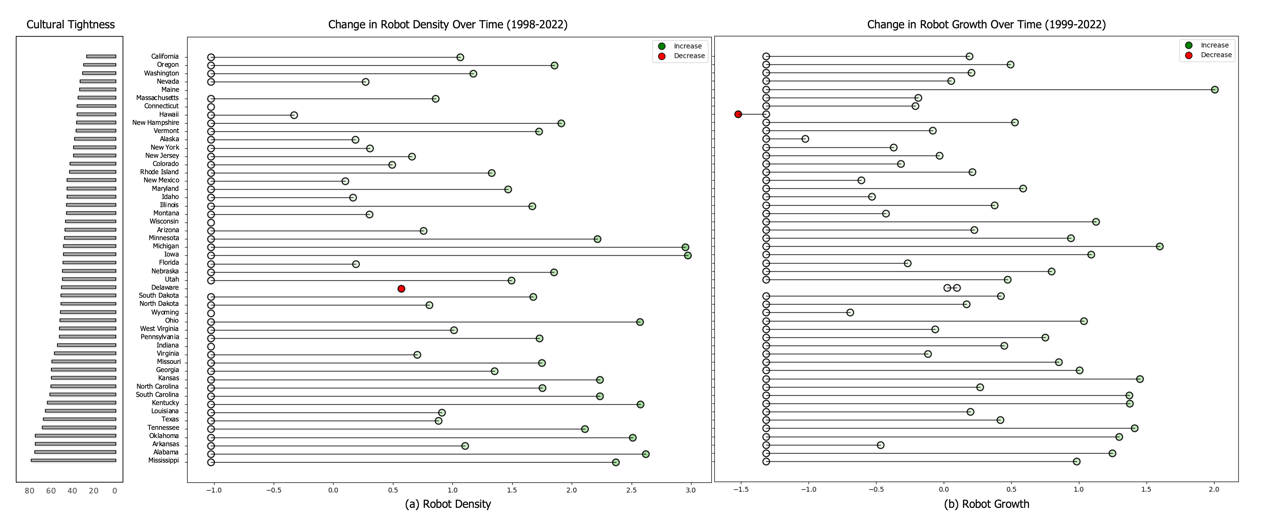
**

**Fig S5A. Culture tightness and robot application across 50 U.S. states from 1998 to 2022 in Study 2a.** Each state is ordered in terms of cultural tightness. White and colored nodes represent the a) robot density for each state and b) robot growth for each state, respectively. Greener nodes represent a greater extent of increase in a) robot density and b) robot growth and red nodes represent declining a) robot density and b) robot growth. All estimates have been standardized via z-scoring. Missing values are replaced by the value of the closest year available.

**
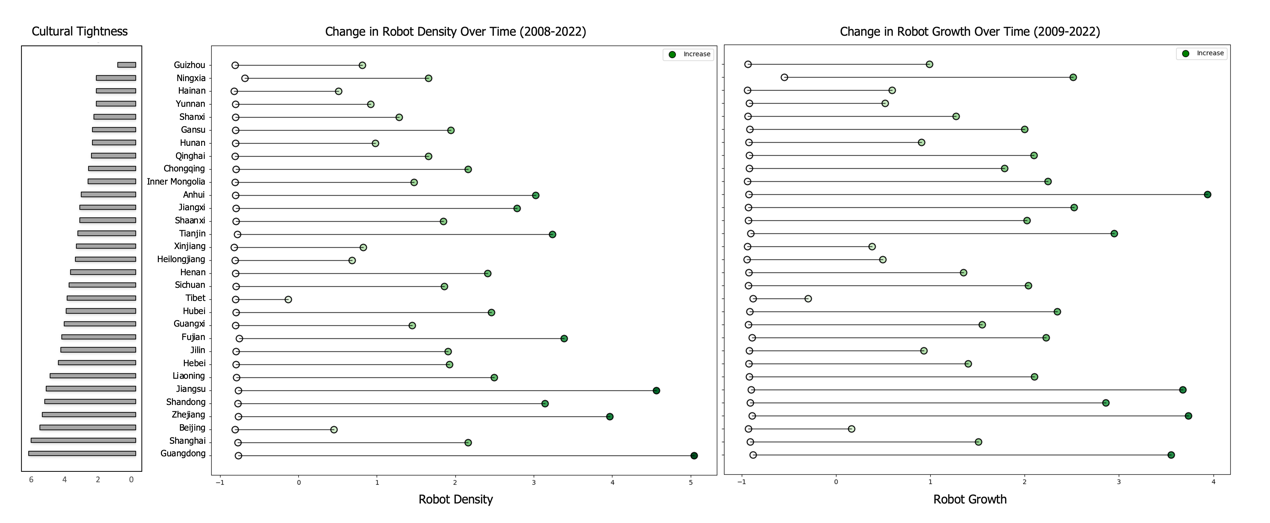
**

**Fig S5B. Culture tightness and robot application across 31 provinces in China from 2008 to 2022 in Study 2b.** Each province is ordered in terms of cultural tightness. White and colored nodes represent the a) robot density for each province, and b) robot growth for each province, respectively. Greener nodes represent a greater extent of increase in a) robot density and b) robot growth and red nodes represent declining a) robot density and b) robot growth. All estimates have been standardized via z-scoring. Missing values are replaced by the value of the closest year available.
