# Supplementary material for: Explaining disparities in robot applications among nations and regions: A cross-level lens of cultural tightness-looseness
Source: PLoS One. 2025 Apr 16;20(4):e0321173. doi: 10.1371/journal.pone.0321173 (PMC12002431; doi:10.1371/journal.pone.0321173)
Supplement: S6 Table — S6A Table. Cultural tightness and robot application at the U.S. state level in Study 2a. S6B Table. Cultural tightness and robot application at the China province level in Study 2b. S6C Table. The interaction effect of cultural tightness and year on robot application at the U.S. state level in Study 2a. S6D Table. The interaction effect of cultural tightness and year on robot application at the China province level in Study 2b. S6E Table. Cultural tightness and robot installation density at the U.S. state level in Study 2a. S6F Table. Cultural tightness and robot installation density at the China province level in Study 2b. (DOCX) [file pone.0321173.s006.docx]

**S6 Table. Additional analyses for Study 2.**

We controlled for spending per GDP and R&D spending per GDP [1] to isolate the effect of these factors on our analyses across 50 states in the United States and across 31 provinces in China. Spending per GDP and R&D spending per GDP at the U.S. state level were collected from the Census Bureau per GDP and the National Science Foundation, respectively. Spending per GDP and R&D spending per GDP at the China province level were collected from the National Bureau of Statistics of China. All findings were replicated, as shown in Table S6A and Table S6B.

**S6A Table. Cultural tightness and robot application at the U.S. state level in Study 2a.**

| **Variables** | **Robot density** | | **Robot growth** | |
| --- | --- | --- | --- | --- |
|  | **Model 1** | **Model 2** | **Model 3** | **Model 4** |
| **Cultural tightness** | 0.11^*^(0.05) | 0.26^**^(0.08) | 0.12^†^(0.07) | 0.23^*^(0.09) |
| **GDP per capita (log)** | –0.09^*^(0.04) | 0.12^*^(0.06) | –0.18^***^(0.06) | –0.04(0.08) |
| **Unemployment rate** |  | –0.05^**^(0.02) |  | –0.09^***^(0.02) |
| **Education spending**  **per GDP** |  | –0.09^**^(0.03) |  | –0.19^***^(0.04) |
| **R&D spending**  **per GDP** |  | –0.01(0.02) |  | –0.01(0.02) |
| **Collectivism** |  | –0.14^†^(0.08) |  | –0.14(0.09) |
| **Constant** | –1.16^***^(0.09) | 1.13^***^(0.13) | 0.69^***^(0.12) | 0.30^†^(0.16) |
| **Observations** | 1188 | 751 | 1115 | 739 |
| **Log Likelihood** | –474.61 | –152.00 | –641.06 | –380.97 |

*Note.* Standard errors in parentheses; All estimates have been standardized via z-scoring for presentation; ^†^ *p* < 0.10, ^*^ *p* < 0.05, ^**^ *p* < 0.01, ^***^ *p* < 0.001.

**S6B Table. Cultural tightness and robot application at the China province level in Study 2b.**

| **Variables** | **Robot density** | | **Robot growth** | |
| --- | --- | --- | --- | --- |
|  | **Model 1** | **Model 2** | **Model 3** | **Model 4** |
| **Cultural tightness** | 0.27^***^(0.07) | 0.13^**^(0.04) | 0.19^*^(0.08) | 0.12^*^(0.05) |
| **GDP per capita (log)** | –0.13(0.09) | –0.28^***^(0.06) | 0.03(0.10) | –0.29^***^(0.08) |
| **Unemployment rate** |  | –0.01(0.02) |  | –0.05(0.03) |
| **Education spending**  **per GDP** |  | –0.08^*^(0.04) |  | –0.06(0.05) |
| **R&D spending**  **per GDP** |  | 0.31^***^(0.04) |  | 0.37^***^(0.05) |
| **Collectivism** |  | –0.05^†^(0.03) |  | –0.09^*^(0.04) |
| **Constant** | –1.00^***^(0.14) | –1.02^***^(0.10) | 1.78^***^(0.14) | 1.64^***^(0.10) |
| **Observations** | 453 | 364 | 420 | 331 |
| **Log Likelihood** | –238.43 | –95.61 | –277.14 | –173.19 |

*Note.* Standard errors in parentheses; All estimates have been standardized via z-scoring for presentation; ^†^ *p* < 0.10, ^*^ *p* < 0.05, ^**^ *p* < 0.01, ^***^ *p* < 0.001.

We adopted Jackson et al.’s [2] approach, employing a longitudinal model with random intercepts and slopes, to test whether cultural tightness was linked to increases in robot application within countries. We found that the interaction between cultural tightness and year on robot density and robot growth within the U.S. (S6C Table, Models 1 and 4) and China (S6D Table, Models 1 and 4) was positively significant. This effect was replicated when we controlled for GDP per capita (U.S.: S6C Table, Model 2s and 5; China: S6D Table, Models 2 and 5) and remained positive when we further controlled for unemployment rate, education spending per GDP, R&D spending per GDP and collectivism (U.S.: S6C Table, Models 3 and 6; China: S6D Table, Models 3 and 6). These findings also provided robust evidence for our hypothesis within countries.

**S6C Table. The interaction effect of cultural tightness and year on robot application at the U.S. state level in Study 2a.**

| **Variables** | **Robot density** | | | **Robot growth** | | |
| --- | --- | --- | --- | --- | --- | --- |
|  | **Model 1** | **Model 2** | **Model 3** | **Model 4** | **Model 5** | **Model 6** |
| **Cultural tightness** | 0.13^**^(0.05) | 0.11^*^(0.05) | 0.22^**^(0.08) | 0.17^**^(0.07) | 0.12^†^(0.07) | 0.22^*^(0.09) |
| **Year** | 0.72^***^(0.02) | 0.78^***^(0.04) | –0.91^*^(0.39) | –2.37^***^(0.61) | –2.03^**^(0.62) | –2.87^***^(0.59) |
| **Cultural tightness × Year** | 0.11^***^(0.01) | 0.11^***^(0.01) | 0.13^***^(0.01) | 0.08^***^(0.01) | 0.07^***^(0.01) | 0.02(0.02) |
| **GDP per capita (log)** |  | –0.06(0.04) | 0.23^***^(0.06) |  | –0.17^**^(0.05) | –0.03(0.08) |
| **Unemployment rate** |  |  | –0.03^*^(0.02) |  |  | –0.09^***^(0.02) |
| **Education spending per GDP** |  |  | –0.03(0.03) |  |  | –0.19^***^(0.04) |
| **R&D spending per GDP** |  |  | –0.01(0.01) |  |  | –0.01(0.02) |
| **Collectivism** |  |  | –0.14^†^(0.08) |  |  | –0.14(0.09) |
| **Constant** | 0.17^**^(0.06) | 0.17^**^(0.06) | 2.52^***^(0.61) | 4.34^***^(0.98) | 4.03^***^(0.98) | 5.06^***^(0.92) |
| **Observations** | 1188 | 1188 | 751 | 1115 | 1115 | 739 |
| **Log Likelihood** | –406.46 | –405.40 | –107.14 | –627.76 | –623.26 | –380.62 |

*Note.* Standard errors in parentheses; All estimates have been standardized via z-scoring for presentation; ^†^ *p* < 0.10, ^*^ *p* < 0.05, ^**^ *p* < 0.01, ^***^ *p* < 0.001.

**S6D Table. The interaction effect of cultural tightness and year on robot application at the China province level in Study 2b.**

| **Variables** | **Robot Density** | | | **Robot Growth** | | |
| --- | --- | --- | --- | --- | --- | --- |
|  | **Model 1** | **Model 2** | **Model 3** | **Model 4** | **Model 5** | **Model 6** |
| **Cultural tightness** | 0.20^***^(0.06) | 0.07(0.07) | 0.07^†^(0.04) | 0.18^**^(0.07) | 0.04(0.08) | 0.06(0.05) |
| **Year** | 0.88^***^(0.03) | 0.69^***^(0.07) | 0.75^***^(0.05) | 1.57^***^(0.45) | 1.44^**^(0.44) | 4.08^***^(0.41) |
| **Cultural tightness × Year** | 0.19^***^(0.02) | 0.21^***^(0.02) | 0.16^***^(0.02) | 0.15^***^(0.02) | 0.17^***^(0.02) | 0.16^***^(0.03) |
| **GDP per capita (log)** |  | 0.27^**^(0.09) | –0.11^†^(0.06) |  | 0.30^**^(0.11) | –0.19^*^(0.08) |
| **Unemployment rate** |  |  | –0.03(0.02) |  |  | –0.06^†^(0.03) |
| **Education spending per GDP** |  |  | –0.07^*^(0.03) |  |  | –0.07(0.05) |
| **R&D spending per GDP** |  |  | 0.26^***^(0.03) |  |  | 0.34^***^(0.05) |
| **Collectivism** |  |  | –0.03(0.03) |  |  | –0.07^†^(0.04) |
| **Constant** | 0.61^***^(0.07) | 0.64^***^(0.07) | 0.40^***^(0.04) | –0.72(0.67) | –0.81(0.67) | –4.12^***^(0.52) |
| **Observations** | 453 | 453 | 364 | 420 | 420 | 331 |
| **Log Likelihood** | –171.18 | –166.30 | –48.35 | –254.01 | –250.26 | –154.40 |

*Note.* Standard errors in parentheses; All estimates have been standardized via z-scoring for presentation; ^†^ *p* < 0.10, ^*^ *p* < 0.05, ^**^ *p* < 0.01, ^***^ *p* < 0.001.

In addition, we measured state-/province-level robot installation density (i.e., installations of industrial robots per 10,000 labor force) as another outcome variable. Since the installations of industrial robots data were only presented by IFR across industries within countries, thus, we first conducted the Bartik IV method to calculate robot installation stock within a region by using Equation 3 in the manuscript. Then we employed Equation 1 to estimate state-/province-level robot installation density. The results showed that cultural tightness was positively related to robot installation density within the U.S. (S6E Table, Models 1–3) and China (S6F Table, Models 1–3), and the interaction between cultural tightness and year also remained significant within the U.S. (S6E Table, Models 4–6) and China (S6F Table, Models 4–6).

**S6E Table. Cultural tightness and robot installation density at the U.S. state level in Study 2a.**

| **Variables** | **Model 1** | **Model 2** | **Model 3** | **Model 4** | **Model 5** | **Model 6** |
| --- | --- | --- | --- | --- | --- | --- |
| **Cultural tightness** | 0.16^**^(0.06) | 0.12^†^(0.06) | 0.27^**^(0.09) | 0.16^**^(0.06) | 0.12^*^(0.06) | 0.25^**^(0.09) |
| **GDP per capita (log)** |  | –0.15^***^(0.04) | 0.08(0.05) |  | –0.12^**^(0.04) | 0.15^**^(0.05) |
| **Unemployment rate** |  |  | –0.03^*^(0.01) |  |  | –0.02^†^(0.01) |
| **Education spending per GDP** |  |  | –0.07^**^(0.03) |  |  | –0.03(0.03) |
| **R&D spending per GDP** |  |  | –0.02(0.01) |  |  | –0.02(0.01) |
| **Collectivism** |  |  | –0.15^†^(0.09) |  |  | –0.15^†^(0.09) |
| **Year** |  |  |  | 0.67^***^(0.02) | 0.78^***^(0.04) | 0.49(0.33) |
| **Cultural tightness × Year** |  |  |  | 0.10^***^(0.01) | 0.09^***^(0.01) | 0.08^***^(0.01) |
| **Constant** | –1.26^***^(0.08) | –1.47^***^(0.10) | 0.81^***^(0.12) | –0.14^*^(0.07) | –0.14^*^(0.07) | –0.08(0.51) |
| **Observations** | 1188 | 1188 | 751 | 1188 | 1188 | 751 |
| **Log Likelihood** | –447.14 | –441.65 | –10.58 | –393.16 | –388.96 | 13.81 |

*Note.* Standard errors in parentheses; All estimates have been standardized via z-scoring for presentation; ^†^ *p* < 0.10, ^*^ *p* < 0.05, ^**^ *p* < 0.01, ^***^ *p* < 0.001.

**S6F Table. Cultural tightness and robot installation density at the China province level in Study 2b.**

| **Variables** | **Model 1** | **Model 2** | **Model 3** | **Model 4** | **Model 5** | **Model 6** |
| --- | --- | --- | --- | --- | --- | --- |
| **Cultural tightness** | 0.23^***^(0.06) | 0.30^***^(0.08) | 0.15^***^(0.04) | 0.22^***^(0.06) | 0.08(0.07) | 0.08^†^(0.04) |
| **GDP per capita (log)** |  | –0.16^†^(0.09) | –0.32^***^(0.06) |  | 0.29^**^(0.09) | –0.12^†^(0.07) |
| **Unemployment rate** |  |  | –0.01(0.03) |  |  | –0.02(0.02) |
| **Education spending per GDP** |  |  | –0.09^*^(0.04) |  |  | –0.09^*^(0.04) |
| **R&D spending per GDP** |  |  | 0.35^***^(0.04) |  |  | 0.29^***^(0.03) |
| **Collectivism** |  |  | –0.06^†^(0.03) |  |  | –0.04(0.03) |
| **Year** |  |  |  | 0.87^***^(0.02) | 0.66^***^(0.07) | 0.84^***^(0.05) |
| **Cultural tightness × Year** |  |  |  | 0.19^***^(0.01) | 0.21^***^(0.02) | 0.16^***^(0.02) |
| **Constant** | –0.92^***^(0.09) | –1.12^***^(0.15) | –1.15^***^(0.10) | 0.50^***^(0.07) | 0.54^***^(0.07) | 0.45^***^(0.04) |
| **Observations** | 453 | 453 | 364 | 453 | 453 | 364 |
| **Log Likelihood** | –227.30 | –226.00 | –90.46 | –158.59 | –153.27 | –44.10 |

*Note.* Standard errors in parentheses; All estimates have been standardized via z-scoring for presentation; ^†^ *p* < 0.10, ^*^ *p* < 0.05, ^**^ *p* < 0.01, ^***^ *p* < 0.001.

**References**

1. Kokko A, Tingvall PG, Videnord J. The Growth Effects of R&D Spending in the EU: A Meta-Analysis. Economics. 2015;9: 20150040. doi:10.5018/economics-ejournal.ja.2015-40

2. Jackson JC, Yam KC, Tang PM, Sibley CG, Waytz A. Exposure to automation explains religious declines. Proc Natl Acad Sci USA. 2023;120: e2304748120. doi:10.1073/pnas.2304748120
